# The mitochondrial ubiquitin ligase MARCHF5 cooperates with MCL1 to inhibit apoptosis in KSHV-transformed primary effusion lymphoma cell lines

**DOI:** 10.1371/journal.ppat.1014665

**Published:** 2026-10-01

**Authors:** Prasanth Viswanathan, Justine R. Bersonda, Jackson Gill, Alyssandra Navarro, Allison C. Farrar, Thomas Caldarera, Andrew Murphy, Daniel Dunham, Karl W. Boehme, Mark Manzano

**Affiliations:** 1 Department of Microbiology and Immunology, University of Arkansas for Medical Sciences, Little Rock, Arkansas, United States of America; 2 Center for Microbial Pathogenesis and Host Inflammatory Responses, University of Arkansas for Medical Sciences, Little Rock, Arkansas, United States of America; 3 Winthrop P. Rockefeller Cancer Institute, University of Arkansas for Medical Sciences, Little Rock, Arkansas, United States of America; 4 Program in Biomedical Engineering, University of Arkansas, Fayetteville, Arkansas, United States of America; 5 Program in Health Sciences and Interdisciplinary Studies, Hendrix College, Conway, Arkansas, United States of America; University of Washington, UNITED STATES OF AMERICA

## Abstract

Kaposi’s sarcoma-associated herpesvirus (KSHV) causes several malignancies in people with HIV including Kaposi’s sarcoma and primary effusion lymphoma (PEL). We have previously shown that PEL cell lines require myeloid cell leukemia-1 (MCL1) to inhibit apoptosis. MCL1 is an oncogene that is amplified in cancers and causes resistance to chemotherapy regimens. MCL1 is thus an attractive target for drug development. The emerging clinical relevance and therapeutic potential of MCL1 motivated us to study the roles of this oncogene in PEL in depth. Using a systems biology approach, we uncovered an unexpected genetic interaction between *MCL1* and *MARCHF5* indicating that they function in the same pathway. MARCHF5 is an E3 ubiquitin ligase best known for regulating mitochondrial homeostasis and antiviral signaling, but not apoptosis. We thus investigated how MCL1 and MARCHF5 cooperate to promote PEL cell survival. CRISPR knockout (KO) of *MARCHF5* in PEL cell lines resulted in a significant increase in apoptosis despite the presence of MCL1. The anti-apoptotic function of MARCHF5 was dependent on its E3 ligase and dimerization activities. Loss of MARCHF5 or inhibition of the 26S proteasome furthermore stabilized the MCL1 antagonist NOXA without affecting levels of MCL1. Interestingly, *NOXA* KO provides a fitness advantage to PEL cells suggesting that NOXA is the pro-apoptotic signal that necessitates the anti-apoptotic activities of MCL1 and MARCHF5. Endogenous reciprocal co-immunoprecipitation experiments show that MARCHF5 and NOXA are found in the same protein complex. Finally, *NOXA* KO rescued the loss of cell viability due to *MARCHF5* KO. Our findings thus provide the mechanistic link that underlies the genetic interaction between MCL1 and MARCHF5. We propose that MARCHF5 induces the degradation of the MCL1 antagonist NOXA thus reinforcing the pro-survival role of MCL1 in these tumor cells. This newly appreciated interaction of the MCL1 and MARCHF5 oncogenes may be useful to improve the design of combination therapies for KSHV malignancies.

## Author summary

Kaposi’s sarcoma-associated herpesvirus (KSHV) is a tumor virus that causes the cancers Kaposi’s sarcoma and primary effusion lymphoma (PEL). We previously identified that PEL tumor cells require the oncogene MCL1 to protect cells from apoptosis. In this study, we uncovered that this function is reinforced by another oncogene, MARCHF5. We found that MARCHF5 is similarly required to protect from apoptosis by causing the turnover of the MCL1 antagonist NOXA. By alleviating the inhibitory effect of NOXA on MCL1, we propose that the oncogenes MCL1 and MARCHF5 cooperate in the same pathway to promote tumor cell survival. Understanding this functional reinforcement may be useful in the future to improve the design of combination therapies for KSHV cancers.

## Introduction

Kaposi’s sarcoma-associated herpesvirus (KSHV) is a human gammaherpesvirus that establishes life-long latency primarily in B cells. While the majority of infections remain asymptomatic, KSHV-positive people with HIV (PWH) can develop several hematologic diseases, including the lymphoproliferative disorder multicentric Castleman disease, and the rare but aggressive primary effusion B cell lymphoma (PEL) [1,2]. PEL classically presents as effusions in body cavities, including the pleural, pericardial, and peritoneal spaces, although extra-cavitary cases have been reported [3,4]. PEL comprises 4–8% of non-Hodgkin lymphoma cases in PWH [5,6]. Currently, there is no standard therapy for PEL. Patients receive similar chemotherapy regimens as with aggressive non-Hodgkin lymphomas (cyclophosphamide, hydroxydaunomycin/doxorubicin, oncovine/vincristine, and prednisolone or CHOP) and anti-retroviral therapy (ART) [1]. Nevertheless, the median overall survival time is 6–22 months [3,5–10].

While mutations in primary PEL tumors have been reported, the majority of PEL cases do not have genetic alterations in the tumor suppressor genes *TP53* or *RB1,* nor do they have *MYC* translocations frequently observed in other non-viral B cell lymphomas [2–4,11–14]. Instead, the major driver of PEL is oncogenic “addiction” or constitutive dependency on host genes that are transcriptionally or post-transcriptionally dysregulated by KSHV genes. In our previous genome-wide CRISPR-Cas9 knockout (KO) screens, we identified 210 PEL-specific oncogenic dependencies (PSODs) or addictions that are preferentially required by PEL cell lines but not by non-PEL cancer cell lines [15]. These PSODs function in viral persistence strategies that overlap with hallmarks of cancer: metabolic rewiring, transcriptional reprogramming, cell cycle progression, sustaining proliferative signaling, immune evasion, and inhibition of cell death. Because of their essential roles in tumor cell survival, PSODs are attractive therapeutic candidates to treat PEL.

At the top of this list was the anti-apoptotic protein myeloid cell leukemia-1 (MCL1), which ranked as the second most essential PSOD [15,16]. *MCL1* is an oncogene that has copy number amplification in solid tumors and hematologic malignancies, including non-Hodgkin lymphoma [17]. Overexpression of MCL1 in different cancers is associated with disease severity and poor prognosis, underscoring the pro-survival role of this oncoprotein [18,19]. With the development of the first-in-class MCL1 inhibitor S63845 [20], new and more potent MCL1 inhibitors are now being evaluated in the clinic [21]. This growing attention to MCL1 as a chemotherapeutic target to treat different cancers initiated our interest in the role of this oncogene in PEL.

MCL1 is a member of the B cell lymphoma-2 (BCL2) protein family that functions to prevent intrinsic apoptosis. BCL2 proteins are anchored to the mitochondrial outer membrane (MOM) to sequester monomers of the cell death effectors BAX or BAK1 [22]. The binding of BCL2 proteins to BAX/BAK1 prevents the monomers from assembling into a pore complex and initiating the apoptosis cascade. During intrinsic apoptosis, pro-apoptotic BCL2 homology region 3 (BH3)-only proteins are upregulated and bind with high affinity to the BCL2 proteins to displace BAX/BAK1. The released BAX/BAK1 molecules oligomerize to form mitochondrial pores and trigger MOM permeabilization (MOMP) [23]. While some BH3-only proteins can bind to all BCL2 family members (e.g., BIM, truncated BID, and PUMA), others are more selective. For instance, NOXA specifically neutralizes MCL1, while HRK has high specificity to BCL-XL.

We previously showed that PEL primary tumors and cell lines highly express MCL1 [15,24]. PEL cell lines are only addicted to MCL1 and do not depend on the other pro-survival BCL2 genes [15]. Consistent with this finding, we and others demonstrated that inhibitors against MCL1, but not other BCL2 proteins, are highly effective in killing PEL cell lines [24–26]. Moreover, the KSHV latency-associated nuclear antigen (LANA) sequesters the MCL1 E3 ubiquitin ligase FWB7, thereby stabilizing the MCL1 protein and promoting tumor cell survival [26]. Together, these observations underscore the role of the oncogenic addiction to MCL1 in this viral lymphoma.

Given the importance of MCL1 in PEL, we sought to uncover how MCL1 is regulated. In this study, we used an unbiased systems biology approach to identify that another high-scoring PSOD, MARCHF5, cooperates with MCL1 function. We show that this mitochondrial E3 ubiquitin ligase targets the MCL1 antagonist NOXA for degradation and consequently reinforces MCL1 function in protecting tumor cells from apoptosis.

## Results

### MCL1 and MARCHF5 are co-essential oncogene addictions in PEL cells

To gain unbiased insights into functions of MCL1, we took a systems biology approach to identify the genetic interactions of MCL1. We reanalyzed publicly available data from the Cancer Dependency Map (DepMap) [27]. DepMap integrates data from CRISPR screens from >1000 human cancer cell lines that identify genes essential for cell fitness to create “co-essentiality” maps. Genes that function in the same pathway have positive interactions because their individual knockout have similar phenotypic effects (co-essential genes or synergists) regardless of context. On the other hand, genes that have opposing functions have antagonistic interactions because the knockout of one negates the phenotype caused by the knockout of the other (antagonists). Because co-essentiality mapping only measures phenotypes and is thus agnostic to prior knowledge of gene function, this approach can lead to the discovery of new gene functions and pathway relationships [27–30].

We examined the co-essentiality maps of *MCL1* from DepMap. Consistent with its known role in preventing intrinsic apoptosis, *MCL1* shows antagonistic interactions with several pro-apoptotic effectors, including *PMAIP1* (encodes for NOXA), *BAX*, *APAF1*, and *BAK1* (Fig 1A, S1 Table). Similarly, *MCL1* exhibits co-essentiality with other members of the BCL2 family, *BCL2L2* and *BCL2* (ranks 3 and 11, respectively)*.* However, the top co-essential gene with *MCL1* was the mitochondrial E3 ubiquitin ligase *MARCHF5*, which had a correlation score that is twice as strong as compared to *BCL2L2 or BCL2*. In addition, the E2 ubiquitin ligase *UBE2J2* ranked second, just before the BCL2 genes. Conversely, *MCL1* was the top co-essential gene of *MARCHF5,* followed by *UBE2J2* and another E2 ubiquitin ligase *UBE2K* (Fig 1B, S1 Table). This relationship is surprising because MARCHF5 is more established as a regulator of mitochondrial homeostasis and antiviral signaling [31–33]. Recent reports, however, have implicated MARCHF5 in the degradation of MCL1 itself or the MCL1-NOXA complex that could result in the inhibition of apoptosis [34,35], facilitation of apoptosis [36] or can also have no effect [37] depending on the context. Regardless, these findings underscore the functional link between MCL1 and this mitochondrial ubiquitin ligase complex.

**Fig 1:**
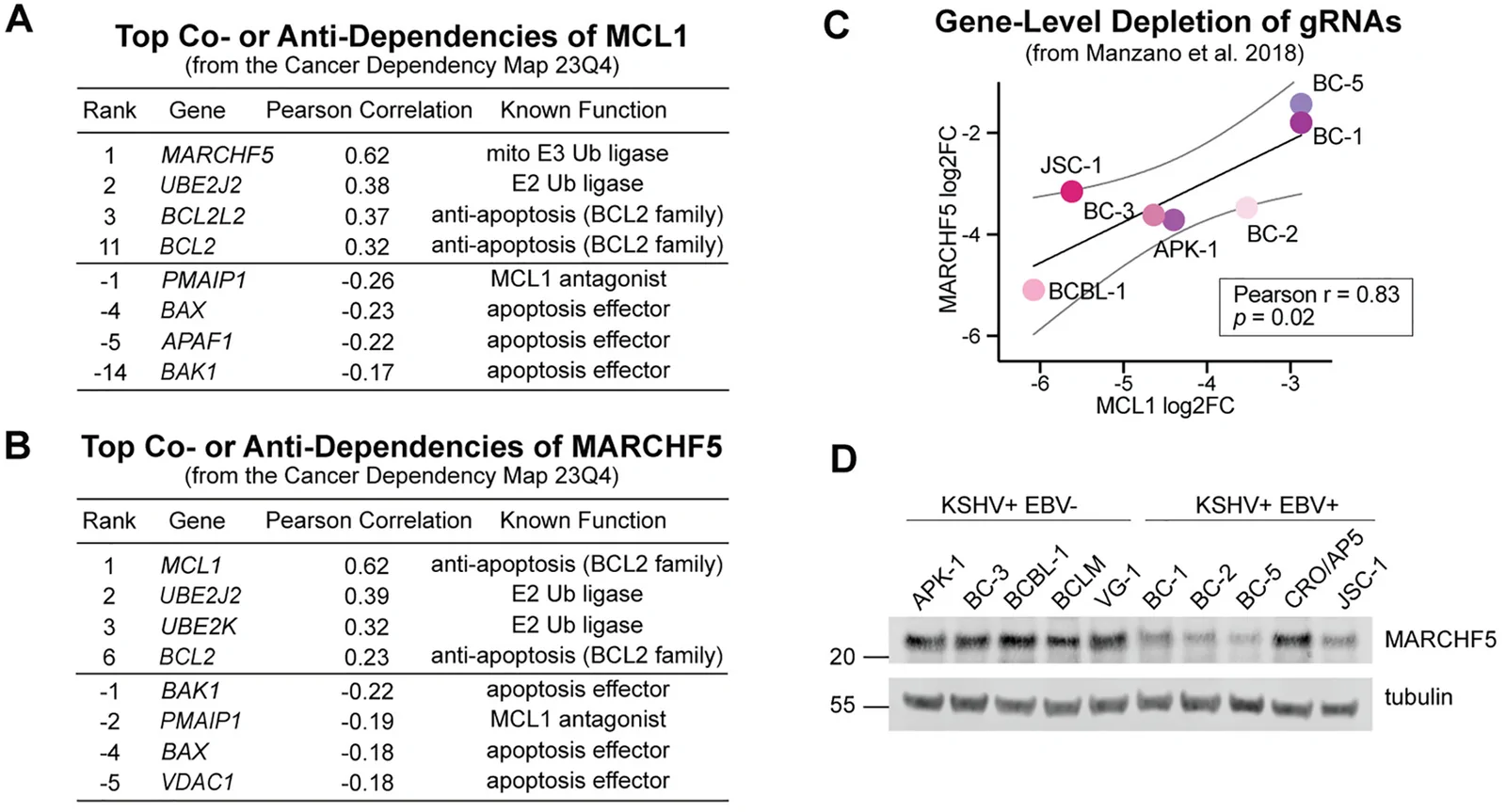
*MARCHF5* is a co-dependent oncogene with *MCL1* in PEL cell lines. A-B. Top co- and anti-dependencies of A. *MCL1* or B. *MARCHF5* from the Cancer Dependency Map 23Q4. The top 100 genes are listed in S1 Table. C. Median fold changes of gRNAs targeting *MCL1* or *MARCHF5* in our gene essentiality CRISPR screens in PEL cell lines [15]. D. Western blots of MARCHF5 in a panel of PEL cell lines that are infected with KSHV only or co-infected by KSHV and EBV.

To investigate if *MCL1* and *MARCHF5* exhibit positive co-essentiality in PEL cell lines, we reexamined our previous CRISPR screens that identify PSODs [15,16]. *MCL1* and *MARCHF5* ranked as the second and third strongest PSOD for PEL cell lines in an updated reanalysis of our previous screens [16]. While these indicate that *MCL1* and *MARCHF5* are dependency factors in PEL, these ranks, by themselves, do not inform potential interactions between these genes as in DepMap.

We therefore calculated the Pearson correlation coefficient of the consolidated depletion scores for guide RNAs (gRNAs) targeting *MCL1* or *MARCHF5* similar to DepMap. These strongly correlated across the individual cell lines, even more consistent than the computed correlation coefficient from DepMap (Pearson *r* = 0.83, Fig 1C). In addition, MARCHF5 protein was expressed at similar levels in a wider set of cell lines (Fig 1D). Together, co-essentiality analyses of CRISPR screens suggests that the two mitochondrial PSODs *MCL1* and *MARCHF5* may cooperate to promote fitness and survival of PEL cell lines.

### MARCHF5 is required for the survival of PEL cell lines

To validate if PEL cell lines are addicted to *MARCHF5* expression, we targeted *MARCHF5* for CRISPR-Cas9 KO using two independent gRNAs in two representative PEL cell lines: BC-3 expressing a constitutive Cas9 [15] and BCBL-1 expressing a doxycycline (Dox)-inducible Cas9 (iCas9) [38]. In both cell lines, *MARCHF5* KO resulted in lower numbers of total live cells compared to the negative gRNA, which targets the safe harbor locus *AAVS1* (Fig 2A–C). CRISPR screens from DepMap independently confirm that *MARCHF5* is a dependency gene in PEL cell lines but not in uninfected non-cancerous immortalized B cells, prostate and retinal pigment epithelial cells (Fig 2D).

**Fig 2:**
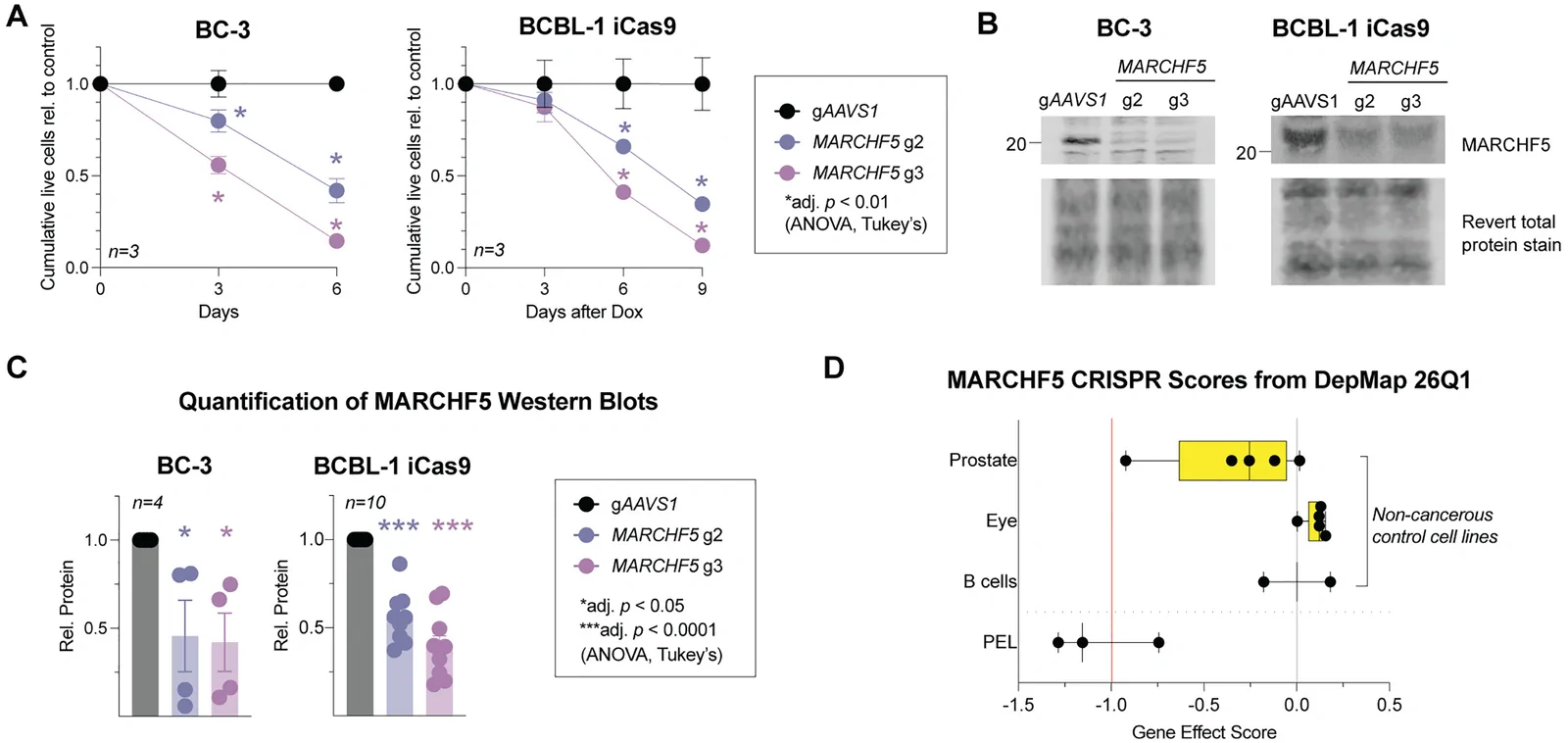
MARCHF5 is required for survival of PEL cell lines. A. Cumulative live cell counts of *MARCHF5* Cas9 knockout (KO) cells. To start the growth curves, BC-3 cells were seeded at 3 x 10^5^ cells/mL 3-4 days after transduction with lentiviruses expressing both Cas9 and the targeting gRNA against *AAVS1* or *MARCHF5*. BCBL-1 iCas9 cells expressing the gRNAs were similarly seeded in triplicates and Cas9 expression was induced by Doxycycline (Dox) treatment (0.5 μg/mL). Every three days, total live cells were counted by trypan blue exclusion assay and split back to 3 x 10^5^ cells/mL to allow the cells to continue proliferating. Cumulative live cell counts are expressed relative to the negative control gRNA *AAVS1*. B. Representative Western blots of MARCHF5 from cells harvested at Day 6 from similar experiments from panel A. C. Quantification of MARCHF5 Western blots normalized to Revert Total Protein stain signal from *MARCHF5* KO cells from experiments collected for this study. For panels A and C, statistical differences were calculated using two-way ANOVA with Tukey’s multiple comparison post-hoc test. Adjusted *p* values reflect post-hoc comparisons between g*AAVS1* to either *MARCHF5* gRNA at the specific time point. Error bars, standard error of the mean. D. *MARCHF5* CRISPR Scores (Gene Effect Score) from genome-wide CRISPR screens in non-cancerous control immortalized cell lines (5 prostate cell lines, 5 retinal pigment epithelial cells, and 2 B cell lines) as well as 3 PEL cell lines were downloaded from DepMap 26Q1. As reference points, a Gene Effect Score of 0 (gray line) denotes that a gene does not affect fitness of a specific cell line while a Gene Effect Score (red line) of 1 represents the median fitness score for pan-essential genes (“housekeeping”) defined by DepMap. The box plots and whiskers represent the median and lower and upper limits of each group.

The co-essentiality of *MARCHF5* and *MCL1* suggests that these mitochondrial proteins are functioning in the same pathway. To test whether MARCHF5 also prevents intrinsic apoptosis like MCL1, we measured the activities of the terminal caspases 3/7 using a luminescence-based Caspase-Glo 3/7 Assay. In both BC-3 Cas9 and BCBL-1 iCas9 cells, *MARCHF5* KO significantly increases caspase 3/7 activities (Fig 3A). Similarly, levels of the early apoptotic marker Annexin V and dead cell stain 7-AAD increased in BCBL-1 *MARCHF5* KO cells (Fig 3B). Taken together, these data confirm that MARCHF5 is a dependency factor in PEL cell lines required to inhibit apoptosis.

**Fig 3:**
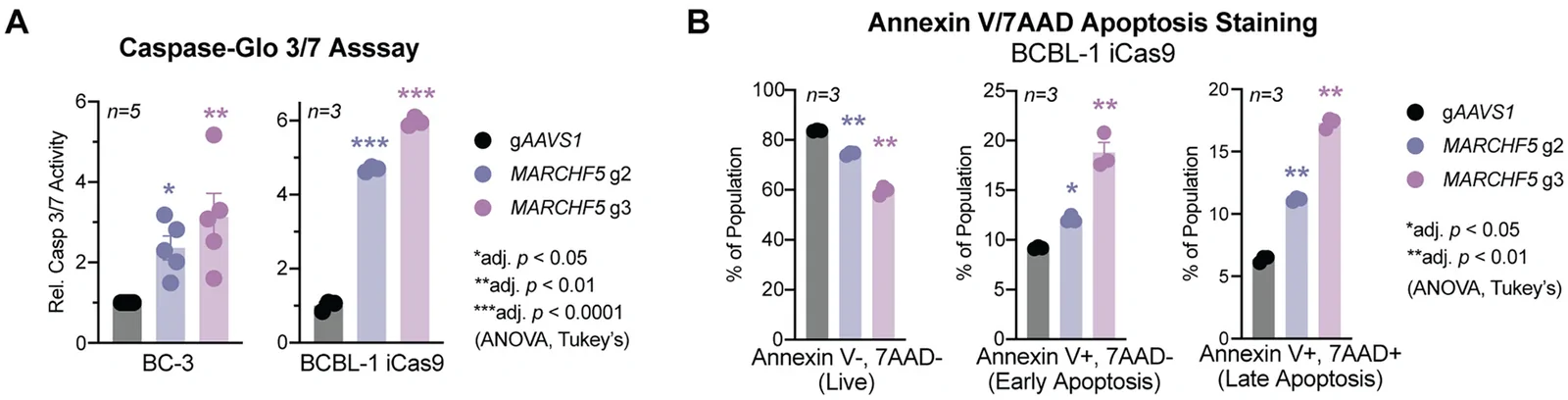
*MARCHF5* KO induces apoptosis. Apoptosis was measured using A. Caspase 3/7 activity using the Caspase-Glo 3/7 Luminescence Assay or B. levels of Annexin V-7/AAD using the FITC Annexin V Apoptosis Detection Kit with 7-AAD for flow cytometry analysis from BC-3 and BCBL-1 iCas9 *MARCHF5* KO cells harvested at Day 6 of the growth curves from Fig 2. Statistical differences were calculated using one-way ANOVA with Tukey’s multiple comparison post-hoc test (n = 3-5). Adjusted *p* values reflect post-hoc comparisons between g*AAVS1* to either *MARCHF5* gRNA. Error bars, standard error of the mean.

### Ubiquitin ligase activity and dimerization of MARCHF5 are required for the survival of PEL cell lines

MARCHF5 is an E3 ubiquitin ligase anchored to the mitochondrial outer membrane [31,32]. It is known to regulate mitochondrial structure and dynamics by ubiquitinating proteins that regulate either mitochondrial fission or fusion. Furthermore, MARCHF5 protein levels are tightly regulated by auto-ubiquitination and homodimerization through GxxxG motifs to maintain proper mitochondrial homeostasis [39].

To determine if dimerization and/or the ubiquitin ligase activity is essential for MARCHF5 function in PEL cells, we transduced BCBL-1 iCas9 cells expressing the *MARCHF5* gRNAs with lentiviruses expressing cDNAs encoding for mCherry (negative control), a positive control 3XFLAG-tagged codon-optimized wild-type MARCHF5 (coMARCHF5 WT), a ligase mutant (coMARCHF5 H43W), or a dimerization mutant (coMARCHF5 4GL) [39,40]. Our codon optimization strategy disrupts the targeting sites for the *MARCHF5* gRNAs, thus restricting editing to only the endogenous alleles.

Surprisingly, these cells did not appear to over-express the coMARCHF5–3XFLAG cDNAs by Western blot (S2A Fig). We hypothesized that over-expressing MARCHF5 increases its auto-ubiquitination [39] thus reducing its steady state levels. To determine if over-expression of MARCHF5 increases its proteasome-mediated turnover, we treated the BCBL-1 g*AAVS1* cells expressing the MARCHF5 cDNA constructs with MG132. S2B Fig shows that this increased MARCHF5 protein expressed from all the cDNAs consistent with an autoregulatory feedback. We thus repeated the Western blots on Dox-induced BCBL-1 iCas9 cells (9 days) treated with MG132 for 8 h prior to harvesting. Fig 4A–B show that Dox-induced editing reduced endogenous MARCHF5 protein but not the 3X-FLAG-tagged cDNA in the presence of MG132.

**Fig 4:**
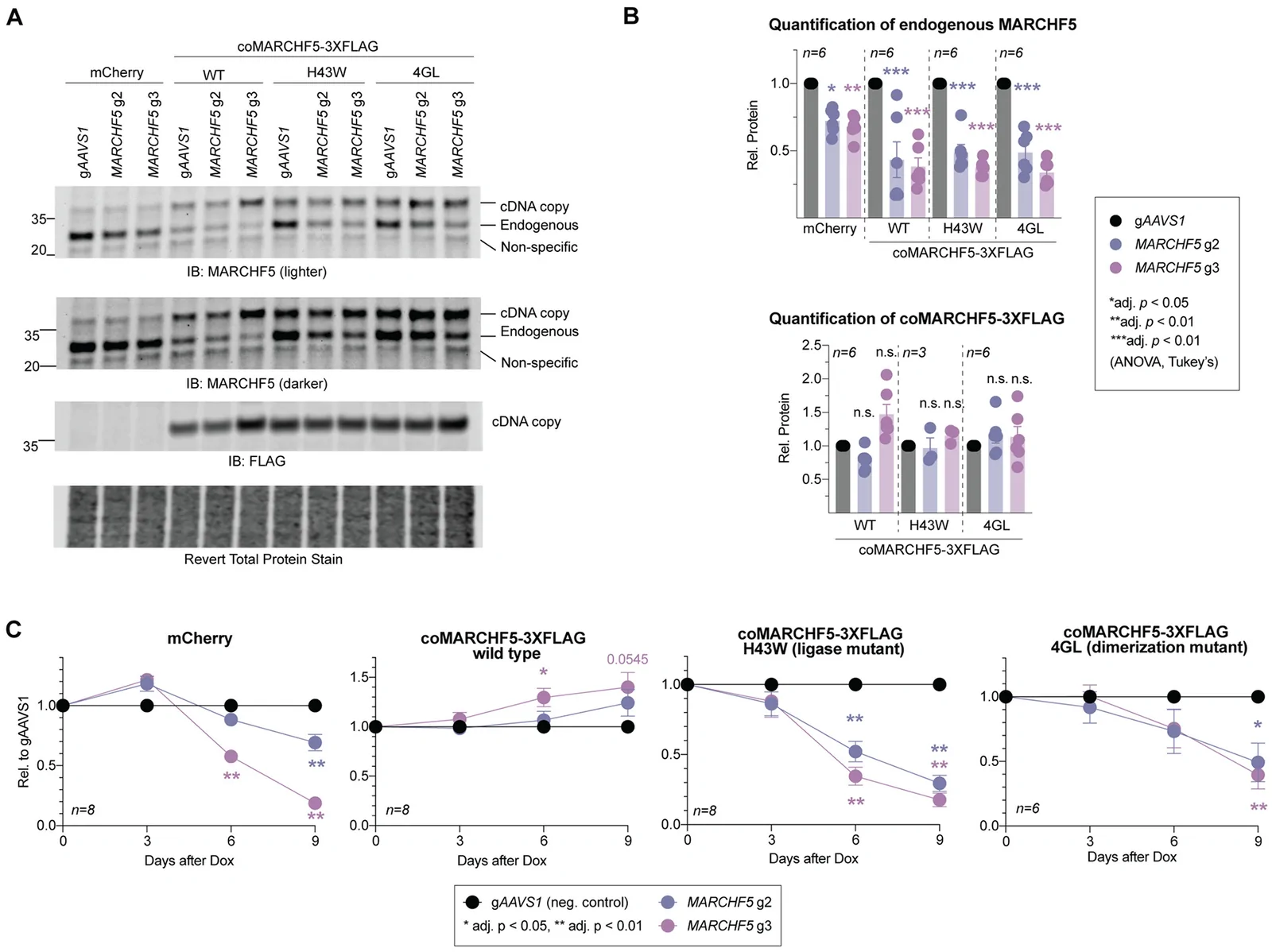
MARCHF5 requires its ubiquitin ligase and dimerization activities in PEL. A. Western blots of endogenous and 3XFLAG-tagged MARCHF5. BCBL-1 iCas9 cells expressing gRNAs targeting *MARCHF5* or *AAVS1* were transduced with lentiviruses constitutively expressing cDNAs for mCherry, codon-optimized MARCHF5 (coMARCHF5) wildtype, H43W ligase mutant, or 4GL dimerization mutant and treated with Doxycycline (Dox) (0.5 μg/mL) for 9 days. To visualize the coMARCHF5 expressed from the lentivirus, cells were treated with 2.5 μM MG132 for 8 hour prior to harvesting for Western blotting. B. Quantification of Western blots in panel A normalized to total protein stain and g*AAVS1.* C. Cumulative live cell counts (relative to control g*AAVS1*) of BCBL-1 iCas9 cells in panel A after the addition of Dox (no MG132). Cells were seeded at 3 x 10^5^ cells/mL and Cas9 expression was induced by Dox treatment (0.5 μg/mL). Every three days, total live cells were counted by trypan blue exclusion assay and split back to 3 x 10^5^ cells/mL to allow the cells to continue proliferating. Cumulative live cell counts are expressed relative to the negative control gRNA *AAVS1*. Statistical differences were calculated using two-way ANOVA with Tukey’s multiple comparison post-hoc test (n = 6-8). Adjusted *p* values reflect post-hoc comparisons between g*AAVS1* to either *MARCHF5* gRNA at the specific time point. Error bars, standard error of the mean.

Nevertheless, the expression of coMARCHF5 WT completely rescued the decrease of total live cells in *MARCHF5* g2 or g3 cells in the absence of MG132 (Fig 4C). This result indicates that the defects on cell fitness are specific to *MARCHF5* KO and not from off-target effects. Moreover, neither the H43W ligase mutant nor the 4GL dimerization mutant rescued *MARCHF5* KO cells despite being expressed at similar levels with the ectopic WT copy. Although the cells expressing the coMARCHF5 WT have lower endogenous MARCHF5 levels (Fig 4A), this did not affect the proliferation of these cells compared to cells expressing mCherry or the coMARCHF5 mutants (S2C Fig). This is likely because the expression of the ectopic coMARCHF5 wt can compensate for the decrease in the endogenous protein. These data collectively show that both the ubiquitin ligase activity and dimerization of MARCHF5 are required for the survival of PEL cell lines.

### Cell death mediated by *MARCHF5* KO is not caused by MAVS activation or lytic replication

One of the described functions of MARCHF5 is to negatively modulate antiviral signaling [33]. Upon sensing viral infections, RIG-I-like receptors (RLRs) are activated and bind to the signaling adapter MAVS on the mitochondrial surface. MAVS consequently transduces RLR signaling by activating antiviral kinases. This eventually leads to the phosphorylation of the transcription factors IRF3 and IRF7 and the transcription of interferon-stimulated genes. MAVS-mediated signaling is kept in check through a negative feedback loop wherein MARCHF5 induces MAVS protein turnover. Because MAVS restricts KSHV lytic replication [41], it is possible that the cell death from loss of MARCHF5 results from altered MAVS signaling and, consequently, the latency-lytic balance.

To test if cell death from *MARCHF5* KO is caused by dysregulated MAVS levels and signaling, we immunoblotted for total MAVS, phosphorylated IRF3 (p-IRF3), and total IRF3. As a control for p-IRF3, we ran protein lysates from 293T cells transfected with the pCDNA vector or pCDNA expressing STING which is well-established to activate IRF3 and promoters of interferon stimulated genes [42,43]. Total MAVS, p-IRF3, and IRF3 remained unchanged in BC-3 *MARCHF5* KO cells while p-IRF3 dramatically increased in STING-transfected 293T cells (Fig 5A). Moreover, we did not detect upregulation of representative early and late lytic proteins (K-bZIP and ORF26, respectively) unlike our positive control lanes for reactivation which are BCBL-1 cells treated with 12-O-tetradecanoylphorbol-13-acetate (TPA) and sodium butyrate (NaB) for 2 days. Similar results were observed in BCBL-1 iCas9 cells (Fig 5B). These data indicate that *MARCHF5* KO-induced apoptosis is not due to MAVS stabilization or aberrant lytic reactivation.

**Fig 5:**
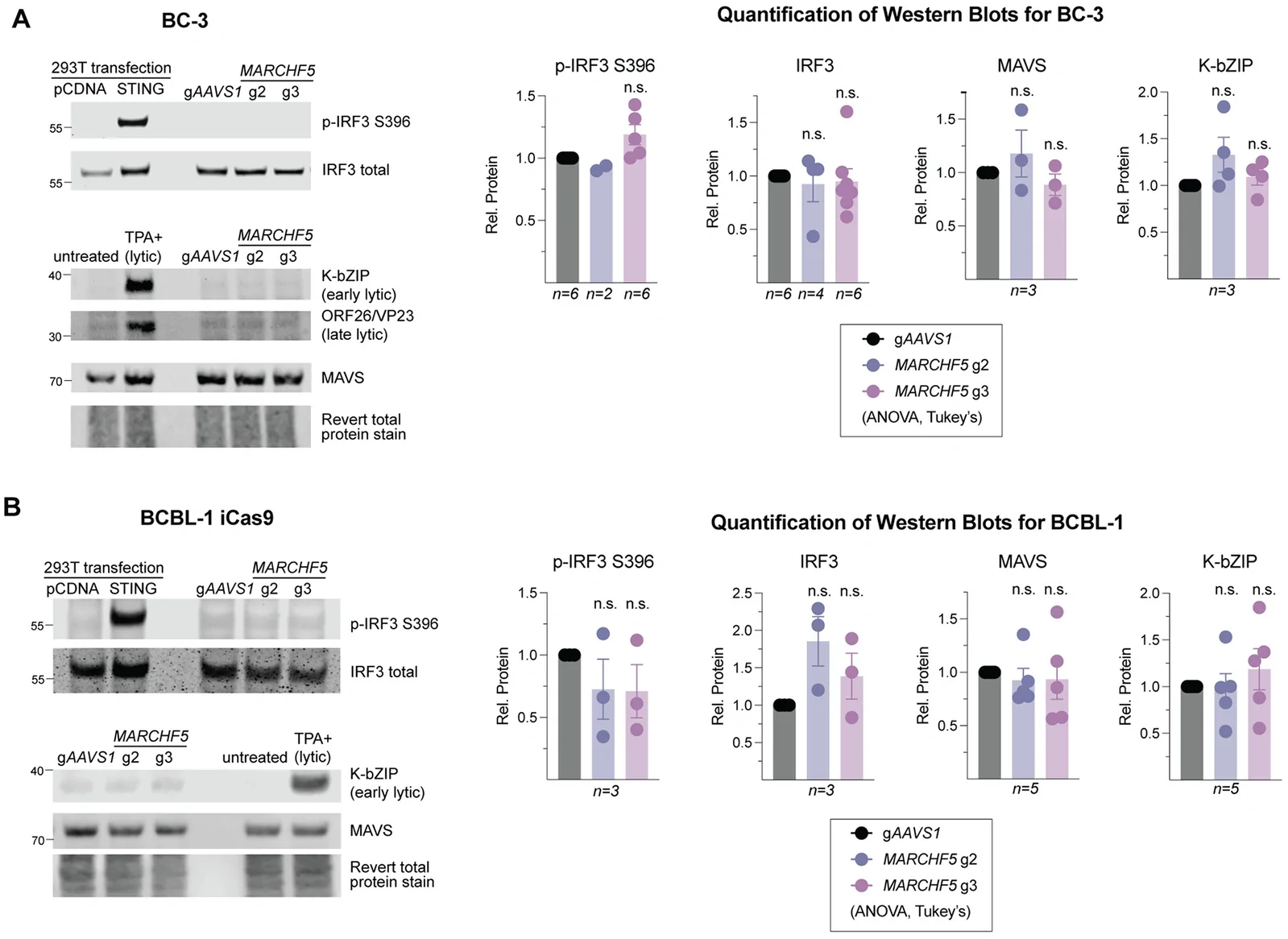
*MARCHF5* KO does not activate the IRF3 signaling pathway nor lytic reactivation. Western blots of proteins involved in KSHV lytic reactivation (K-bZIP) or activation of the IRF3 signaling pathway (MAVS, phospho-IRF3) in A. BC-3 and B. BCBL-1 iCas9 *MARCHF5* KO cells harvested at Day 6 of the growth curves. BCBL-1 PEL cells treated with 20 ng/mL 12-O-tetradecanoylphorbol-13-acetate (TPA) and 1 mM sodium butyrate (NaB) for 2 days serve as a positive control for viral reactivation. 293T cells transfected with an expression construct for human STING for 2 days serve as a positive control for phospho-IRF3. Quantification of Western blots are shown on the right normalized to total protein stain and g*AAVS1*. Statistical differences were calculated using two-way ANOVA with Tukey’s multiple comparison post-hoc test. Adjusted *p* values reflect post-hoc comparisons between g*AAVS1* to either *MARCHF5* gRNA. Error bars, standard error of the mean.

### NOXA restricts the steady-state survival of PEL cell lines

Thus far, our current and previous work [15,24] has shown that PEL cells exhibit oncogenic co-dependencies to MARCHF5 and MCL1 to prevent intrinsic apoptosis, independent of inhibition of MAVS signaling or lytic reactivation. However, it remains unclear how they are mechanistically connected. MARCHF5 has been reported to ubiquitinate MCL1 itself to induce apoptosis [36] or target the MCL1-NOXA complex to inhibit cell death [34,35]. Because both MARCHF5 and MCL1 are pro-survival dependency factors in PEL cell lines, it is unlikely that MARCHF5 ubiquitinates MCL1 for degradation as this will induce apoptosis. We hypothesize that MARCHF5 prevents apoptosis by instead targeting the MCL1 antagonist NOXA for degradation (Fig 6A), consistent with previous reports [34,35]. Consistent with this, co-dependency interactions from DepMap reveal that both *MCL1* and *MARCHF5* are strongly anti-correlated with *PMAIP1* (NOXA) which supports our hypothesis that MARCHF5 ubiquitinates an MCL1 antagonist for degradation (Fig 1A-B, S1 Table).

**Fig 6:**
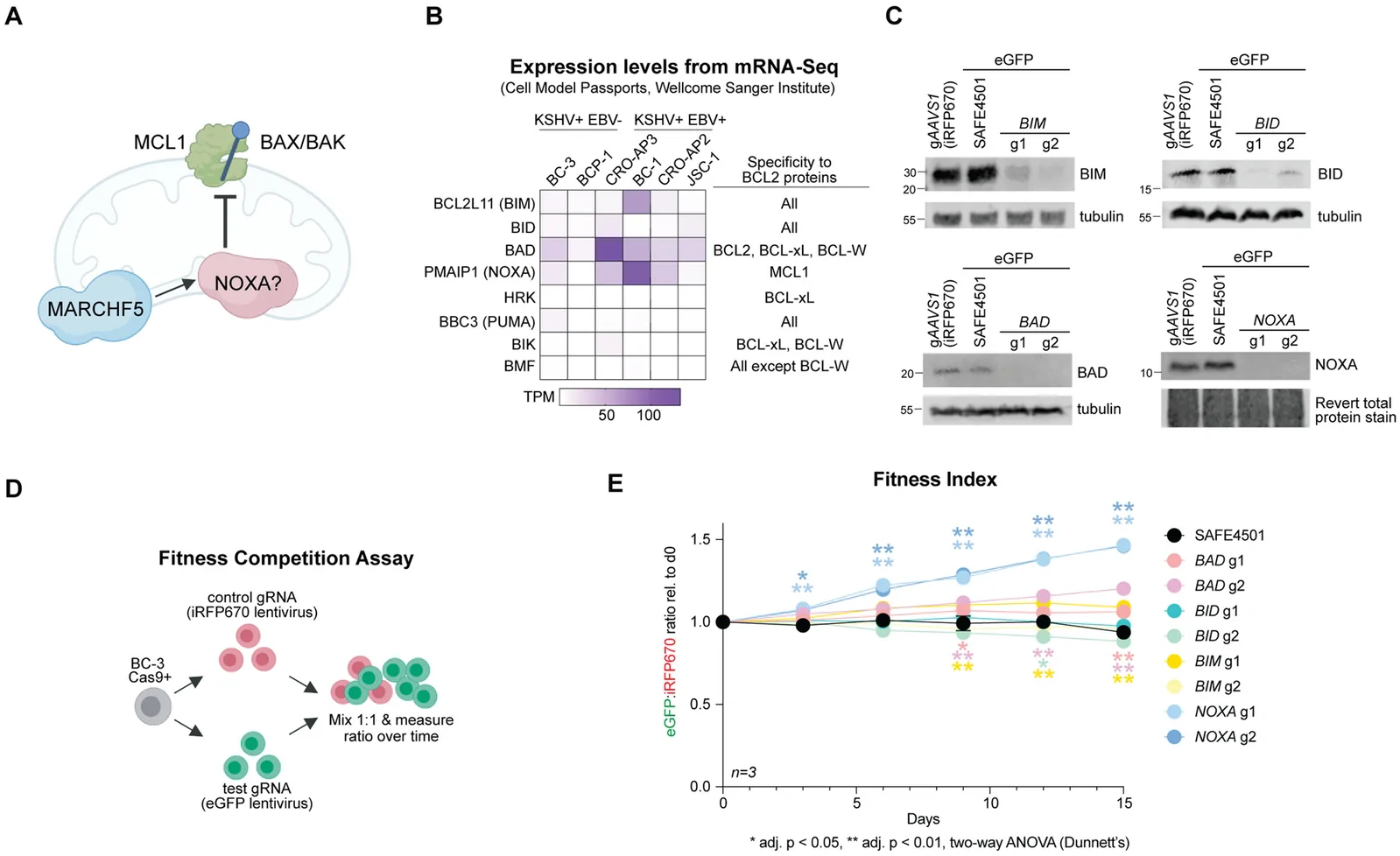
NOXA is a constitutive pro-apoptotic signal in PEL cell lines. A. Model to link the oncogenic functions of MARCHF5 and MCL1. We propose that in PEL cell lines, MARCHF5 cooperates with MCL1 function to induce the proteasomal turnover of the MCL1 antagonist NOXA. B. mRNA expression of BH3-only proteins in PEL cell lines from Cell Model Passports. C. Western blots of BH3-only proteins that were targeted for Cas9 KO in BC-3 cells. D. Experimental flow chart of the Fitness Competition Assay. BC-3 Cas9 cells were transduced with a lentivirus encoding the control g*AAVS1* and an iRFP670 fluorescent protein or a second lentivirus encoding the targeting gRNA and an eGFP reporter. Equal amounts of control iRFP670 cells were mixed with targeted eGFP cells at the beginning of the growth curve. Fitness indices as reflected by the ratio of eGFP to iRFP670 (eGFP:iRFP670) were measured over 15 days by flow cytometry. E. Fitness indices for each BH3-only KO competition relative to day 0. Statistical differences were calculated using two-way ANOVA with Dunnett’s multiple comparison post-hoc test (n = 3). Adjusted *p* values reflect post-hoc comparisons between gSAFE4501 to BH3-only protein gRNA. Error bars, standard error of the mean.

To confirm whether NOXA is the apoptotic trigger in PEL cells that necessitates the anti-apoptotic activities of MCL1 and MARCHF5, we measured the relative fitness of BC-3 cells with wildtype or *NOXA* KO. We hypothesized that Cas9 KO of the apoptotic trigger would provide a fitness advantage to these tumor cells. Although MCL1 is specifically neutralized by NOXA, other promiscuous pro-apoptotic BH3-only proteins can also bind to MCL1 (Fig 6B). To get an unbiased picture of the apoptotic trigger(s) in PEL cells, we extended our analysis to other related BH3 only proteins regardless of their specificity to MCL1. We included the top four BH3-only genes that had measurable basal RNA expression in different PEL cell lines: *BCL2L11* (BIM), *BID*, *BAD*, and *PMAIP1* (NOXA) (Fig 6B). We targeted each of these genes for KO in BC-3 Cas9 cells using two independent test gRNAs expressed from a lentivirus encoding an eGFP fluorescence reporter (Fig 6C). As a negative control, we used the SAFE4501 gRNA that targets a non-functional, non-genic region [44]. In parallel, we derived another negative control cell line expressing the g*AAVS1* gRNA from a lentivirus encoding an iRFP670 fluorescence reporter.

Given the extensive adaptation of cancer cell lines during tumor development in patients and prolonged culture in vitro, we anticipated that the fitness advantage from single-gene KOs would be modest due to redundant or compensatory pathways that optimize cellular fitness. We performed a Fitness Competition Assay (FCA) instead of the standard cell counting method we used in Fig 2. We chose this approach because the FCA provides greater sensitivity and is less susceptible to variability introduced during counting and cell splitting of multi-day growth curves. In the FCA, eGFP+ cells expressing the test gRNA are mixed at a 1:1 equal ratio with the negative control iRFP670 + cells expressing g*AAVS1* (Fig 6D). Relative fitness of the KO cells can be monitored using the ratio of eGFP to iRFP670 as a readout by flow cytometry.

Of the eight test KO cell lines, *NOXA* KO cells consistently had a significant fitness advantage for both gRNAs over the negative control cells gSAFE4501 and g*AAVS1* (Fig 6E). While both *BAD* KO and *BIM* KO cell lines had statistically significant changes, these effects are minor and can only be seen in one gRNA. These experiments suggest that NOXA is the major constitutive apoptotic signal that restricts the steady state survival of PEL cell lines.

### MARCHF5 is in the same complex as the MCL1 antagonist NOXA

To test if NOXA is constitutively regulated at the protein level to promote the survival of PEL cells, we treated PEL cell lines with the proteasome inhibitor MG132. MG132 treatment dramatically stabilized NOXA between 2-fold (BC-3 and JSC-1) to 4-fold (BCBL-1) (Fig 7A, B). In contrast, no changes in MARCHF5 levels were observed while MCL1 was marginally but consistently stabilized ~1.5-fold in all three cell lines. This is consistent with a previous report that MCL1 is regulated by the E3 ubiquitin ligase FWB7 in PEL cells [26] although this does not exclude that it can still be ubiquitinated by MARCHF5. This indicates that the NOXA and, to some extent MCL1, undergo rapid turnover by the proteasome at steady-state in all three PEL cell lines.

**Fig 7:**
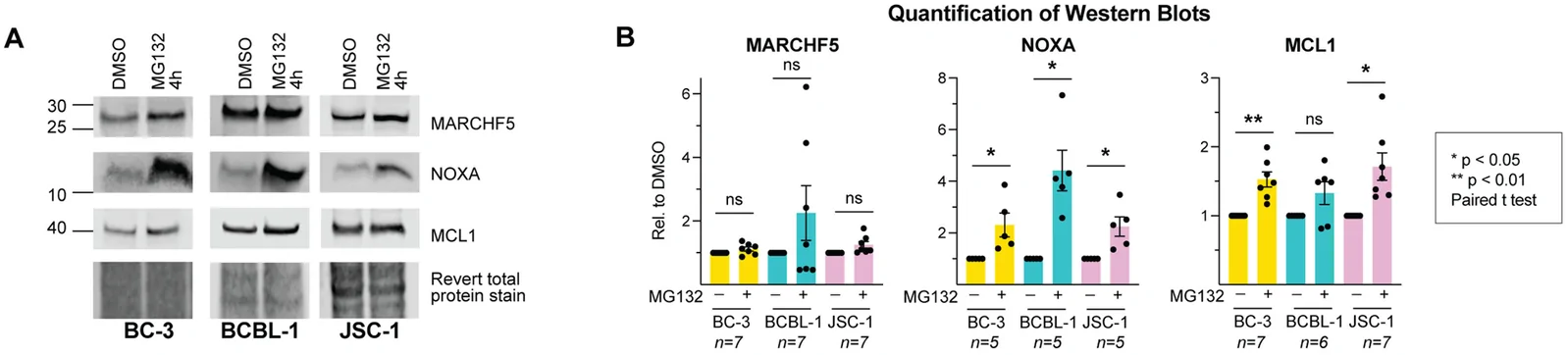
NOXA stability is regulated by the proteasome. A. Western blots of PEL cell lines were treated with DMSO or 1.25 μM MG132 for 4 hours. B. Quantification of Western blots in panel A normalized to total protein stain and DMSO-treated cells. Statistical differences were calculated using paired t tests between untreated and MG132 treated in each cell line. Error bars, standard error of the mean.

To demonstrate that MARCHF5 facilitates NOXA turnover, we immunoblotted for NOXA in *MARCHF5* KO cells. *MARCHF5* KO cells had higher NOXA similar to MG132 treatment alone while MCL1 levels remained statistically unchanged (Fig 8A–B). In contrast, qRT-PCR did not reveal any major increases in *NOXA* mRNA for either cell line (Fig 8C). Moreover, treatment of *MARCHF5* KO cells with MG132 did not further increase total NOXA protein (Fig 8D–E) suggesting that this E3 ligase is involved in the proteasomal turnover but not the transcriptional regulation of NOXA.

**Fig 8:**
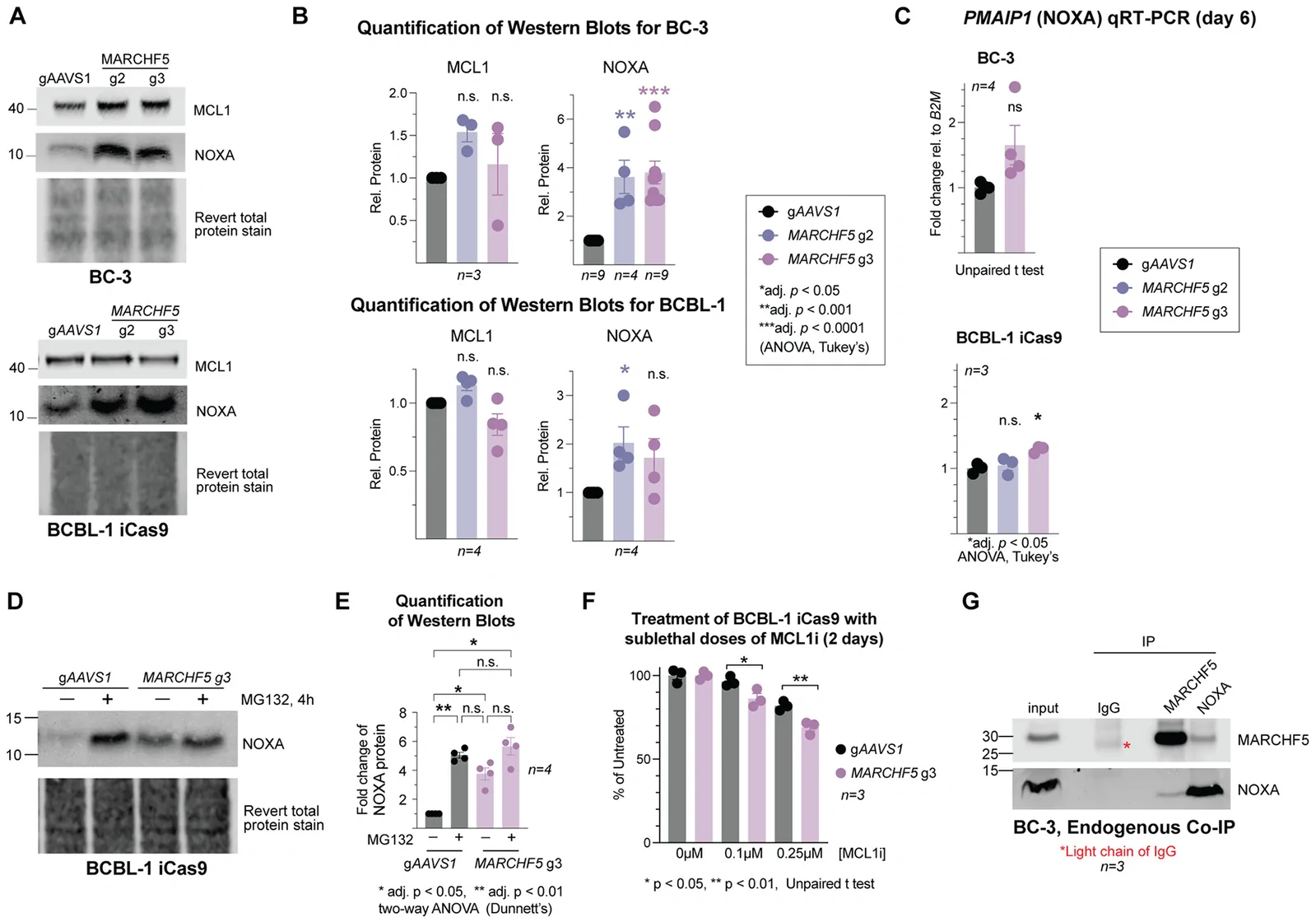
*MARCHF5* KO leads to the stabilization of NOXA protein but not mRNA. A. Western blot for MCL1 and NOXA in wildtype or *MARCHF5* KO BC-3 or BCBL-1 iCas9 cells after 6 days of editing. B. Quantification of Western blot in panel A normalized to total protein stain and g*AAVS1*. C. qRT-PCR of *NOXA* mRNA in wildtype or *MARCHF5* KO BC-3 or BCBL-1 iCas9 cells after 6 days of editing. D. BCBL-1 iCas9 g*AAVS1* or *MARCHF5* g3 were first Dox treated for 9 days to induce editing. Cells were then treated with either DMSO of 1.25 μM MG132 for 4 h before harvesting for Western blotting. E. Quantification of Western blots in panel D normalized to total protein stain and g*AAVS1*. F. Day 9 Dox-treated BCBL-1 iCas9 g*AAVS1* or *MARCHF5* g3 cells were treated with DMSO, 0.1 μM, or 0.25 μM of the MCL1 inhibitor S63845 for 2 days. Cell viability was measured with CellTiter-Glo 2.0 kit and expressed relative to g*AAVS1*. G. Reciprocal co-immunoprecipitation (co-IP) of the endogenous MARCHF5-NOXA complex in BC-3 cells. Statistical differences were calculated using two-way ANOVA with Dunnett’s multiple comparison post-hoc test or unpaired *t* test as noted. Adjusted *p* values reflect post-hoc comparisons between each gRNA to g*AAVS1* unless specifically noted. Error bars, standard error of the mean.

We next asked whether loss of MARCHF5 makes PEL cells more dependent on MCL1 since *MARCHF5* KO should increase NOXA protein. In line with this hypothesis, *MARCHF5* KO cells showed a modest but significant increase in sensitivity to the MCL1 inhibitor S63845 (Fig 8F, MCL1i).

To determine if MARCHF5 directly interacts with NOXA, we performed co-immunoprecipitations (co-IP). IP of endogenous MARCHF5 complexes in BC-3 cells pulled down NOXA while reciprocal co-IP of NOXA complexes conversely pulled down MARCHF5 (Fig 8G). These biochemical experiments confirm that MARCHF5 and NOXA form complexes in PEL cells.

### *NOXA* KO rescues the loss of viability and fitness in *MARCHF5* KO cells

Finally, to demonstrate that MARCH5 and NOXA are functionally connected, we targeted both genes for Cas9 KO. We initially attempted this in BC-3 Cas9 cells so we can measure the fitness effect of *MARCHF5-NOXA* double KO (DKO) in the FCA. However, we were unsuccessful in performing sequential editing in these cells. We therefore used another PEL cell line BC-1 Cas9 which we previously established and validated for efficient editing [15,38].

We first transduced the BC-1 Cas9 cells with puromycin-resistant eGFP lentiviruses expressing the SAFE4501 or *NOXA* g1 gRNAs (Fig 9A). After confirming *NOXA* KO by Western blotting (Fig 9B), we again transduced the SAFE4501 or *NOXA* g1 cells with hygromycin-resistant lentiviruses expressing the g*AAVS1*, *MARCH5* g2 or g3 gRNAs (no fluorescent reporter). After 7 days of selection with hygromycin and confirmation by Western blotting (Fig 9C), we split the cells into three groups: one to harvest for direct measurement with CellTiter-Glo 2.0, another for Caspase-Glo 3/7 Assay and a third to repeat the FCA with BC-1 Cas9 iRFP670 g*AAVS1* cells. After two days, *MARCHF5* KO in the *NOXA* KO background had increased cellular viability and fitness as measured by higher CellTiter-Glo 2.0 signals and eGFP:iRFP670 ratios, but also reduced caspase 3/7 activities when compared to the *MARCHF5* KO in the negative control SAFE4501 gRNA cells (Fig 9D–F). We note that while we can fully rescue cellular viability and fitness as measured by CellTiter-Glo 2.0 and FCA (Fig 9D and F, SAFE4501-*gAAVS1* vs. *NOXA* g1-*MARCHF5* g2), we only saw partial rescue for both readouts in cells with the more efficient *MARCHF5* g3 gRNA (by Western blot). Moreover, while *NOXA* KO significantly reduced the caspase 3/7 activities in both *MARCHF5* g2 and g3 cells (Fig 9E, from 5-fold to 1.5-fold for *MARCHF5* g2, and 3-fold to 2-fold in *MARCHF5* g3), the caspase 3/7 activities remain ~1.5-2X slightly above baseline (Fig 9E, SAFE4501-g*AAVS1* vs. *NOXA* g1-*MARCHF5* g2/3). Together, our data suggests that the major function of MARCHF5 is to induce the degradation of NOXA. In addition, the partial rescues in *NOXA* g1-*MARCHF5* g3 cells indicates that MARCHF5 may have other essential functions in these viral tumor cells although these are likely minor relative to its roles in NOXA regulation.

**Fig 9:**
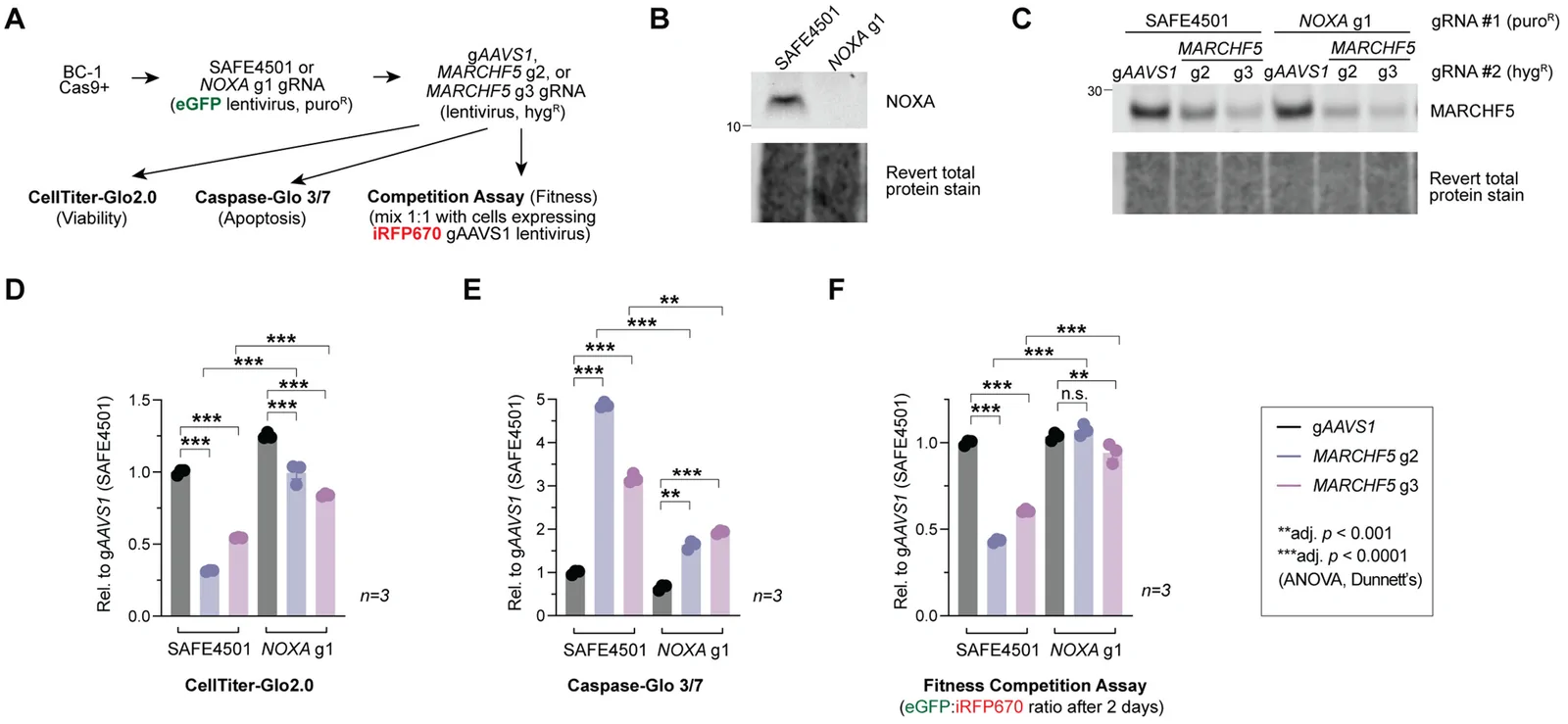
*NOXA* KO restores cellular viability and decreases apoptosis during *MARCH5* KO. A. Workflow of the characterization experiments of the *NOXA-MARCHF5* double KO (DKO) BC-1 Cas9 cells. Cells were transduced with a lentivirus encoding the targeting gRNA (SAFE4501 or *NOXA* g1), an eGFP reporter, and puromycin resistance gene (puro^R^). After selection and confirmation of *NOXA* KO by Western blotting (panel B), these cells were transduced with a second lentivirus encoding another targeting gRNA (g*AAVS1*, *MARCHF5* g2 or g3) and the hygromycin resistance gene (hyg^R^). After 7 days of selection (200 µg/mL) and confirmation of *NOXA* KO by Western blotting (panel C), cell densities were adjusted to 4 x 10^5^ cells/mL and seeded for CellTiter-Glo2.0 Assay, Caspase-Glo 3/7 Assay and FCA for 2 days. B. Western blot of NOXA in SAFE4501 or *NOXA* g1 cells. C. Western blot of NOXA in *MARCHF5*-*NOXA* DKO cells. D-F. DKO cells were characterized using D. CellTiter-Glo2.0 (viability), ***E***. Caspase-Glo 3/7 Assay (caspase 3/7 activity), or F. FCA (fitness). Caspase-Glo3/7 luminescence signals were normalized to CellTiter-Glo2.0 signals to normalize for total viable cells. All data in panels D-F, were further normalized to the SAFE4501-g*AAVS1* DKO cells to establish the baseline. Statistical differences were calculated using one-way ANOVA with Dunnett’s multiple comparison post-hoc test (n = 3). Error bars, standard error of the mean.

In summary, our data support the model that MARCHF5 cooperates with MCL1 by promoting the proteasomal degradation of the MCL1 antagonist NOXA.

## Discussion

In this study, we sought to uncover the genetic interactions of the anti-apoptotic protein *MCL1*, a clinically relevant oncogene in multiple cancers and KSHV-driven PEL [15,17–21,24]. Co-essentiality mapping from DepMap and our own CRISPR screens in PEL cell lines unexpectedly identified that *MCL1* exhibits the strongest genetic interaction with the mitochondrial-resident E3 ubiquitin ligase *MARCHF5*, despite MARCHF5 not being known for being a canonical regulator of apoptosis. Strikingly, *MCL1* and *MARCHF5* are ranked back-to-back as the second and third strongest PSODs in PEL cell lines, underscoring this co-dependency. We find that the amount of MCL1 protein is insufficient to protect from cell death because single CRISPR KO of *MARCHF5* also results in apoptosis. Mechanistically, we find that MARCHF5 induces the turnover of NOXA protein thereby reinforcing the pro-survival role of MCL1 in these tumor cells.

The dependence on both *MCL1* and *MARCHF5* oncogenes underscores that PEL cell lines constantly require NOXA to be kept in check to protect from cell death. The link between MARCHF5 and MCL1 in apoptosis is now being appreciated but is context specific. In prostate cancer, their roles appear to be antagonistic. *MCL1* is amplified in ~10% of cases while *MARCHF5* is deleted in up to ~5% of cases with a higher frequency in metastatic prostate cancers suggesting that they function as an oncogene or tumor suppressor, respectively [36]. Loss of *MARCHF5* in pancreatic cancer cell lines leads to a decrease in dependence in MCL1 consistent with the negative regulation of MCL1 by MARCHF5. This is in sharp contrast to our data in PEL where *MARCHF5* KO cells become more dependent to MCL1 and thus sensitive to MCL1 inhibitors. This mirrors other reports that show a synthetic lethal effect between *MCL1* and *MARCHF5* consistent with a cooperative function between these proteins [34,45]. In this anti-apoptotic role, MARCHF5 has been shown to facilitate the turnover of the MCL1-NOXA complex [35,36]. However, we do not see this in PEL cells. *MARCHF5* KO results in pronounced stabilization of NOXA but not MCL1 in both BC-3 and BCBL-1 cells (Fig 8A–B). Interestingly, MG132 treatment of BC-3 and JSC-1 leads to a small but statistically significant stabilization of the MCL1 protein (Fig 7A–B). This modest stabilization of MCL1 in MG132-treated PEL cells is likely mediated by other E3 ubiquitin ligases because we do not see similar increases of MCL1 protein in *MARCHF5* KO cells. Our data thus point to a different mechanism by which MARCHF5 regulates NOXA separately from the MCL1-NOXA complex.

It remains unclear why NOXA is maintained at steady-state levels that necessitates the addictions to both *MCL1* and *NOXA*. It is worth noting that most PEL cell lines have functional p53 and low but detectable activated p53 [13,46]. It is possible that latent infection and/or replicative stress induced by proliferating tumor cells causes this low-level activation of p53. Thus, p53-responsive genes like *NOXA* need to be controlled by other mechanisms such as MARCHF5-mediated degradation of NOXA and the LANA-mediated stabilization of MCL1 [26]. Interestingly, KSHV encodes for a lytic viral homolog of the BCL2 family (vBCL2) [47,48]. Biochemical studies demonstrate that vBCL2 most resembles MCL1 in sequence and function [49]. Thus, it appears that a major strategy of KSHV to protect infected cells from cell death is to reinforce MCL1 activity or to bring in an MCL1-like protein.

Our study does not rule out that MARCHF5 may have other essential functions in PEL cells. It is possible that the addiction to MARCHF5 also involves the regulation of mitochondrial dynamics to meet the altered energy demands of these actively proliferating tumor cells. Moreover, MARCHF5 has recently been linked to mediating both peroxisome biogenesis and turnover [50,51]. Given that peroxisome proteins are induced by the latency genes and are required for survival by KSHV-infected endothelial cells [52], it is also possible that the dependency to MARCHF5 facilitates peroxisomal metabolism. However, these functions are difficult to observe in PEL due to the confounding effects of apoptosis in subcellular structures. Future studies on the role of MARCHF5 in mitochondrial or peroxisome homeostasis in PEL cell lines should therefore be done in the absence of the apoptotic machinery such as using *BAX/BAK1* double knockout cells.

Finally, the co-essential relationship of MCL1 and MARCHF5 may be exploited for combination therapy to treat PEL. While MCL1 inhibitors or proteasome inhibitors effectively kill PEL cell lines *in vitro* and *in vivo* [15,24,26,53], their use as monotherapies has limitations in the clinic. Two recent Phase I trials using MCL1 inhibitors show that these treatments result in elevated cardiac troponin and cardiovascular risk in patients [54,55]. In addition, although bortezomib is being used to treat multiple myeloma patients, it also induces lytic reactivation in PEL cell lines [53,56] which may pose complications in PWH. By targeting the MCL1-MARCHF5 axis using combination therapy with MCL1 inhibitors and bortezomib, it is possible that a synergistic effect can be achieved at lower doses. More effective treatments will likely prevent the development of resistance and induce apoptosis faster before the virus can complete the lytic cycle. In addition, the lower doses may prevent dose-dependent cardiotoxicity associated with MCL1 inhibitors. Thus, factoring co-essentiality interactions of oncogenes in rational therapy design for KSHV malignancies may improve therapeutic strategies for these cancers.

## Materials and methods

### Cells and treatments

All cloning was performed in *E. coli* Stbl3 cells (Invitrogen, Carlsbad, CA). PEL cell lines were maintained in RPMI-1640 Medium with L-glutamine and sodium bicarbonate (Millipore-Sigma, St. Louis) supplemented with 10% or 20% Serum Plus II Medium Supplement (Millipore-Sigma), 10 μg/mL gentamicin (Gibco, Waltham, MA), and 0.05 mM β-mercaptoethanol (Millipore-Sigma). HEK293T cells were cultured in DMEM with L-glutamine and 4.5 g/L glucose (Millipore-Sigma) supplemented with 10% Serum Plus II Medium Supplement and 10 μg/mL gentamicin. To induce editing in BCBL-1 iCas9, cells were treated with 0.5 μg/mL doxycycline until the end of the growth curve. To induce lytic reactivation, cells were treated with 20 ng/mL TPA and 1 mM sodium butyrate for 2 d. To inhibit proteasome activity, cells were treated with 1.25 μM MG132 for 4 h or 2.5 μM MG132 for 8 h.

### Primers and synthetic genes

All primers were purchased from Integrated DNA Technologies (Coralville, IA). Synthetic genes were purchased from Twist Biosciences (South San Francisco, CA). Sequences are found in S2 Table.

### gRNAs

Primer sequences and cloning strategies for specific gRNA constructs are found in S2 Table. gRNAs for the constitutive Cas9 backbone were cloned using annealed primers into BsmBI-linearized lentiGuide-Puro (a gift from Feng Zhang; Addgene catalog no. 52963) or pLenti SpBsmBI sgRNA Hygro (a gift from Rene Maehr; Addgene catalog no. 62205). gRNAs for iCas9 were cloned into pLX-sgRNA (a gift from Eric Lander and David Sabatini; Addgene catalog no. 50662) as before [38]. To clone gRNAs targeting the BH3-only proteins, we modified the pRDA122_mKate backbone (a gift from the Genetic Perturbation Platform at the Broad Institute). First, the eGFP cDNA was PCR amplified from pLCE [57] using primers 229 and 230. Then, the eGFP PCR product or codon-optimized iRFP670 synthetic gene was cloned into a AgeI/BamHI-linearized pRDA122_mKate. The resulting gRNA vectors pRDA122_eGFP and pRDA122_iRFP670 now express eGFP and iRFP670, respectively, instead of mKate. The negative control gRNA SAFE4501 [44] was cloned into pRDA122_iRFP670. The negative control g*AAVS1* and the rest of the targeting gRNAs were cloned into pRDA122_eGFP. Control g*AAVS1* in pLenti SpBsmBI sgRNA Hygro was previously cloned [58].

### cDNA cloning

All cDNAs used were cloned by Gibson assembly into pLC-coMCL1-IRES-hyg [24] using the NheI/BamHI restriction sites. pLC-mCherry-IRES-hyg is a gift from Eva Gottwein and was previously published [24]. coMARCHF5–3XFLAG cDNA was synthesized by Twist Biosciences. Site-directed mutagenesis were performed to construct the coMARCHF5–3XFLAG mutants using the pLC-coMARCHF5–3XFLAG WT-IRES-hyg as a template (S2 Table). pCDNA3.1 human STING plasmid is a gift from Dr. Megan Orzalli.

### Lentiviruses and lentiviral infection

5 x 10^6^ HEK293T cells were plated in 10 cm dishes. The next day, the medium was replaced with DMEM without any supplements. 5 μg of transfer plasmid, 2.44 μg of pMD2.G (a gift from Didier Trono; Addgene catalog no. 12259) and 3.74 μg of pSPAX2 (a gift from Didier Trono; Addgene catalog no. 12260) were transfected with 39.13 μL of 1 mg/mL polyethylenimine (Sigma-Aldrich, 25kDa Mw branched, pH 7.4). After 4–6 h, media was replaced with complete RPMI-1640 with 10% Serum Plus II Medium Supplement. Cells were grown in the cell culture incubator for three days and viruses were harvested from the supernatant by filtering through a 0.45 μm polyethersulfone membrane.

Cells were either directly infected with undiluted lentiviral supernatants or spinfected with concentrated lentivirus at 1600 *g* for 45 min. After 24 h, cell culture media was replaced with fresh media with antibiotic selection (1 μg/mL puromycin or 100–250 μg/mL hygromycin).

### Growth curves

3 x 10^5^ cells were seeded in 1 mL in 12-well plates. Cells were counted by trypan blue exclusion using the Countess II Automated Cell Counter (Thermo Fisher) every 3 d. Cell densities were adjusted back to 3 x 10^5^ cells/mL to ensure that the cells did not undergo cell cycle arrest.

### Competition assay

We first generated our single BH3-only protein KO in BC-3 or BC-1 Cas9 cells using a lentivirus expressing the targeting gRNA or a negative control gRNA SAFE4501. This lentivirus expresses the fluorescent reporter eGFP in its backbone. In parallel, we also engineered our control competitor BC-3 or BC-1 Cas9 cell line by transducing it with a second lentivirus expressing gAAVS1 and the fluorescent reporter iRFP670 instead of eGFP. After puromycin selection and confirmation of KO by Western blots, each eGFP + KO cell line was mixed at 1:1 with iRFP670 + cells in 12-well plates. To establish an accurate baseline of eGFP:iRFP670 ratios, cell pools were analyzed by flow cytometry at this initial time point. Cells were then grown similar to our Growth Curve Assays and eGFP:iRFP670 ratios were continually monitored every 3 d for 15 d.

### Caspase 3/7 assay

Twenty microliters of resuspended cells were dispensed into 96-well half-area microplates (Greiner Bio-One, Monroe, NC). An equal volume of reconstituted Caspase-Glo 3/7 Reagent (Promega, Madison, WI) was added to each sample and mixed by repeated pipetting. Samples were incubated in the dark for 30 min. Luminescence was read in a Synergy H1 microplate reader (Biotek, Winooski, VT) with an integration time of 2 s.

### Annexin V/7-AAD staining

7 x 10^5^ cells were pelleted and washed twice in 0.5 mL 2% BSA in PBS. The cell pellets were resuspended in 100 μL Annexin V binding buffer (Biolegend, San Diego, CA) with 5 μL Annexin V-APC and/or 5 μL 7-AAD. Cells were stained in the dark for 15 min at 4℃. After incubation, an additional 350 μL of Annexin V binding buffer was added. Samples were analyzed on the BD LSR Fortessa.

### Western blots

Cells were lysed on ice for 10 min with radioimmunoprecipitation assay (RIPA) buffer (20 mM Tris-HCl at pH 7.5, 150 mM NaCl, 1 mM EDTA, 1 mM EGTA, 1% IGEPAL CA-630, 1% sodium deoxycholate) supplemented with protease inhibitor cocktail III (Millipore) and Simple Stop 2 Phosphatase Inhibitor Cocktail (GoldBio, St. Louis, MO). Lysates were clarified by centrifuging at 12,000 g for 10 min at 4℃. Total protein concentrations from clarified lysates were determined using the Pierce BCA Protein Assay Kit (Thermo Scientific). Equal amounts of total protein were loaded and separated in bis-tris acrylamide gels in MES-SDS Running Buffer (Invitrogen) at 100 V. The proteins were transferred to a 0.2 µm nitrocellulose membrane in Towbin buffer (25 mM Tris, 192 mM glycine, 20% methanol, pH 8.3) for 1 h and 45 min at a constant current of 400 mA. Membranes were airdried, stained with Revert 700 Total Protein Stain (LICOR), and imaged using Odyssey Fc. Western blots were blocked with Intercept (TBS) blocking buffer (LICOR) diluted 1:2 in tris-buffered saline (TBS; 20 mM Tris, 150 mM NaCl, pH 7.6) or 5% BSA in TBS for phosphorylated proteins for 1 h. The membranes were incubated with the primary antibody in TBS supplemented with 0.1% Tween-20 (TBST) overnight at 4℃ (See S2 Table). The next day, the membranes are washed 5x with TBST for 10 min each before incubating with IR800CW secondary antibody (LICOR) for 1 h. Final washes in TBST were done before imaging on the Odyssey M (LICOR). Uncropped Western blots are in S1 Fig.

### Reciprocal co-immunoprecipitation (co-IP)

For each co-IP reaction, 2 x 10^7^ BC-3 cells were washed thrice with phosphate-buffered saline (PBS, 137 mM NaCl, 2.7 mM KCl, 1.47 mM KH_2_PO_4_, 8.1 mM Na_2_HPO_4_, pH 7.4) then lysed with 600 μL IP buffer (25 mM HEPES, 100 mM NaCl, 10 mM CaCl_2_, 5 mM MgCl_2_) freshly supplemented with 1% digitonin and protease inhibitor cocktail III. To 400 μL of clarified lysate, IP antibodies were added and incubated on a rotisserie overnight at 4℃: 3.5 μL anti-NOXA antibody (0.55 mg/mL), 4.5 μL anti-MARCHF5 antibody (0.21 mg/mL) or 0.77 μL rabbit IgG (2.5 mg/mL). The remaining lysates were stored at -80℃ as input. The following day, 8 μL of Protein A/G magnetic beads (Pierce) were added to each IP and reincubated on a rotisserie for 4.5 h at 4℃. The beads were washed twice with 500 μL IP buffer with 0.1% digitonin and resuspended in 45 μL 1.5X LDS loading buffer (40 mM Tris-Cl, 53 mM Tris Base, 0.75% lithium dodecyl sulfate, 3.75% glycerol, 0.2 mM EDTA, 3.75% β-mercaptoethanol, pH 8.5). For controls, 27 μL of input was mixed with 9 μL 4X LDS loading dye. All samples were denatured at 90℃ for 7 min. 36 μL of input and 20 μL of each IP were run in each lane of a 12% bis-tris acrylamide gel in MES-SDS Running Buffer at 80V. Western blots for MARCHF5 or NOXA were performed as above.

### RNA isolation

Cells were lysed in 750 μL TRIzol Reagent (Invitrogen) and phase separated with 150 μL chloroform by centrifugation at 12,000 g for 15 min at 4℃. The aqueous phase was transferred to a new tube and mixed with 375 μL 100% isopropanol to precipitate the RNA. The samples were incubated for 10 minutes at room temperature before centrifuging for 10 min at 12,000 g, 4°C. The RNA pellets were washed with 0.75 mL of 75% ethanol before resuspending in pre-warmed nuclease-free water (>60℃).

### DNase treatment

6 μg total RNA was treated with 2U TURBO DNase (Invitrogen) in 1X TURBO DNase Buffer for 30 minutes at 37℃. Reactions were stopped by precipitation of the DNase with the addition of DNase Inactivation Reagent and incubating for 5 min at room temperature. The RNA-containing supernatant was separated and transferred to a new tube after centrifugation at 10,000 g for 3 min.

### cDNA synthesis

cDNA synthesis was done using the High-Capacity cDNA Reverse Transcription Kit (Applied Biosciences). Briefly, 1 μg DNase-treated RNA was first combined with 2 μL 10X RT Random Hexamers and incubated at 65°C for 5 min then flash cooled on ice. Each of the RNA-hexamer mixes were mixed in a total of 20 μL reaction volume with 1X RT Buffer, 4 mM dNTPs, 40 U RNase Inhibitor, and either 50U MultiScribe Reverse Transcriptase or nuclease-free water (for no RT control). All samples were incubated at 10 minutes at 25°C, 2 hours at 37°C, and 5 minutes at 85°C in a thermocycler.

### qPCR

The cDNA or RT- control (0.5 μL) was combined with 2X LUNA Universal qPCR Master Mix (New England Biolabs) and 0.5 μM each of primers (797 and 798 for *NOXA*; 743 and 744 for *B2M* control) in a 20 μL reaction (See S2 Table). Reactions were run in the QuantStudio 6 Pro (Applied Biosystems) under the following conditions: 95°C for 2 min, 40 cycles of 95°C for 15 s, 60°C for 15 s, 72°C for 1 min (data collection step), followed by the default Melt Curve protocol. Ct values and Melt Curve analyses were calculated by QuantStudio 6 Pro using default settings. Fold changes were calculated using 2^-(ΔΔCt)^ method with the *B2M* as the reference gene.

### MCL1i treatments

BCBL-1 iCas9 gAAVS1 or MARCHF5 g3 cells were first treated with Dox for 9 days to induce editing. Cells were then seeded in triplicates in 96-well U-bottom plates at 5000 cells/well and were treated with DMSO only, 0.1 μM, or 0.25 μM of the MCL1 inhibitor S63845 (MCL1i) in a total volume of 150 μL. After 2 days, 20 μL of cells were harvested and mixed with 20 μL of CellTiter-Glo 2.0 (Promega) to measure relative cellular viabilities. Luminescence was read as above.

## Supporting information

S1 Table The Top 100 Codependencies of MCL1 and MARCHF5 downloaded from the Cancer Dependency Map 23Q4. (XLSX)

S2 Table List of sequences of primers, synthetic genes, and antibodies used in this study. Also included are the strategies for cloning for the expression or gRNA vectors that are used in the experiments.
(XLSX)

S1 Fig Uncropped Western blots from this study. (PDF)

S2 Fig MARCHF5 protein levels are autoregulated. A. Western blots of endogenous and 3XFLAG-tagged coMARCHF5 without MG132 treatment from the same growth curves in Fig 4C. BCBL-1 iCas9 cells expressing gRNAs targeting *MARCHF5* or *AAVS1* were transduced with lentiviruses constitutively expressing cDNAs for mCherry, codon-optimized MARCHF5 (coMARCHF5) wildtype, H43W ligase mutant, or 4GL dimerization mutant and treated with Doxycycline (Dox) (0.5 μg/mL) for 9 days and harvested for Western blotting. B. Unedited BCBL-1 iCas9 g*AAVS1* cells expressing mCherry and coMARCHF5–3XFLAG constructs (no Dox) were left untreated or treated with 2.5 μM MG132 for 8 hours prior to harvesting for Western blotting of endogenous and 3XFLAG-tagged coMARCHF5. C. Cumulative live cell counts from the same growth curves of edited BCBL-1 iCas9 g*AAVS1* cells in Fig 4C were replotted relative to the matched mCherry cells to compare the impact of the cDNAs on cellular viability. Statistical differences were calculated using two-way ANOVA with Tukey’s multiple comparison post-hoc test (n = 6–8). Adjusted *p* values reflect post-hoc comparisons between mCherry cells to the other coMARCHF5 constructs at the specific time point. Error bars, standard error of the mean.
(TIF)
