# Supplementary material for: The mitochondrial ubiquitin ligase MARCHF5 cooperates with MCL1 to inhibit apoptosis in KSHV-transformed primary effusion lymphoma cell lines
Source: PLoS Pathog. 2026 Oct 1;22(10):e1014665. doi: 10.1371/journal.ppat.1014665 (PMC13649115; doi:10.1371/journal.ppat.1014665)

Uncropped blots for Figure 1D

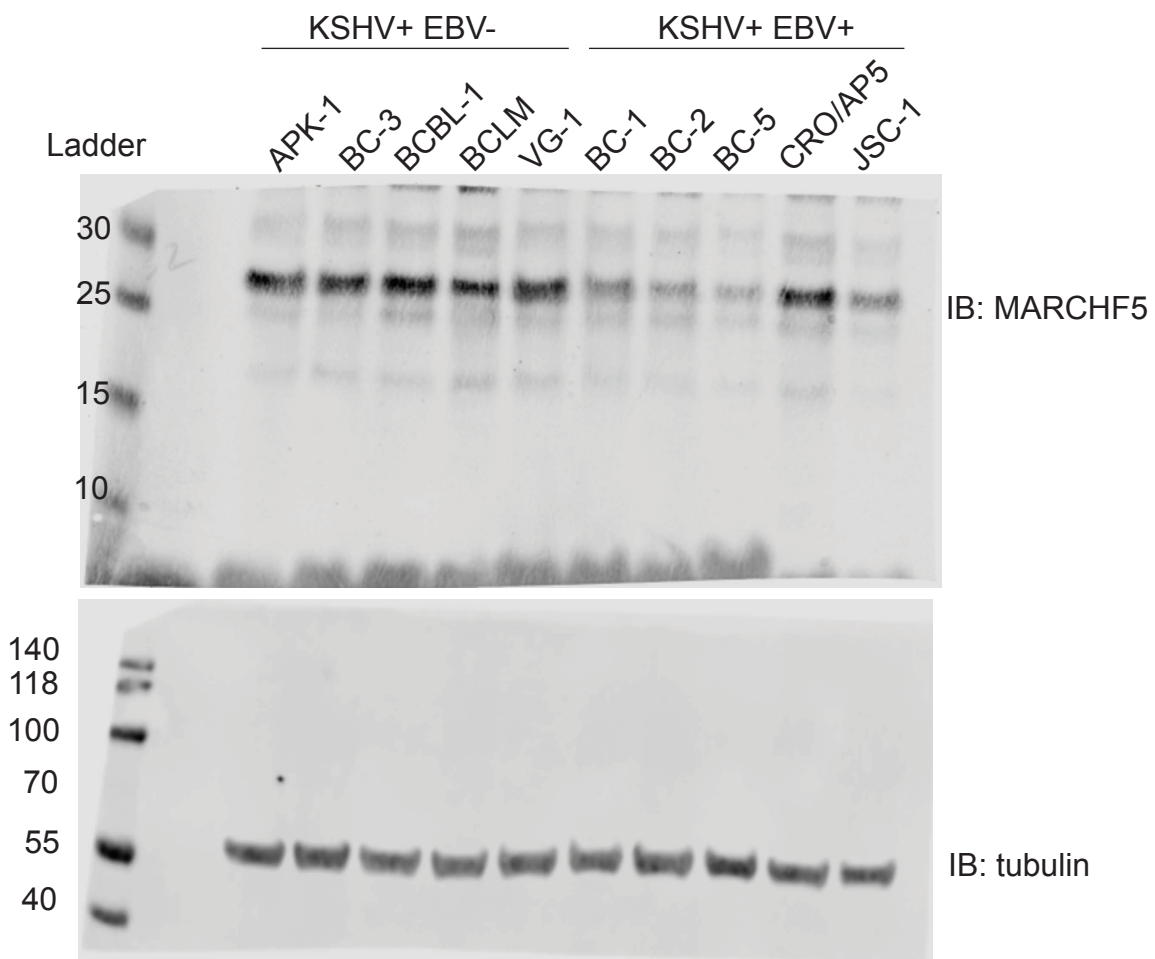

Uncropped blots for Figure 2B, BC-3

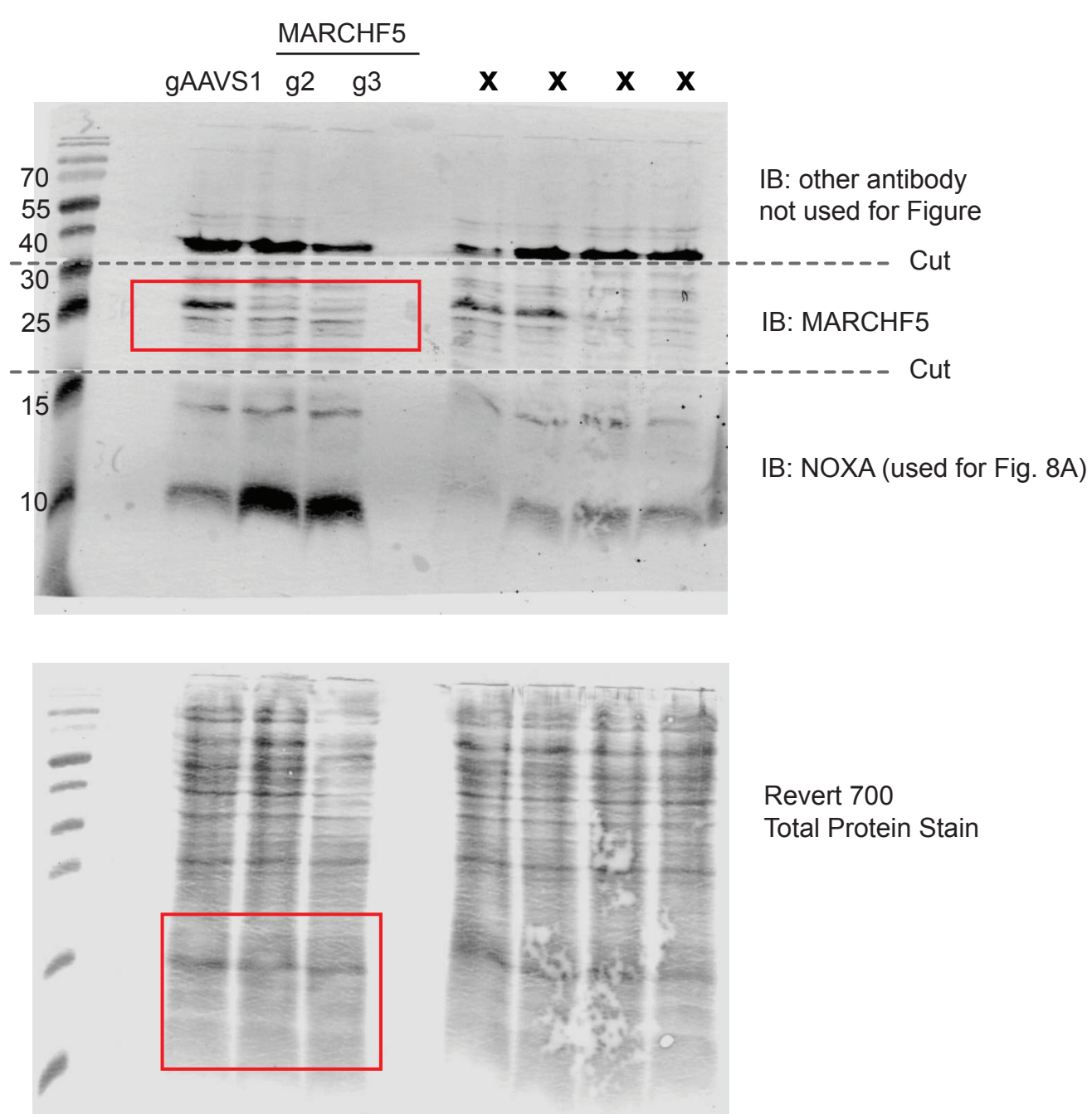

Uncropped blots for Figure 2B, BCBL-1

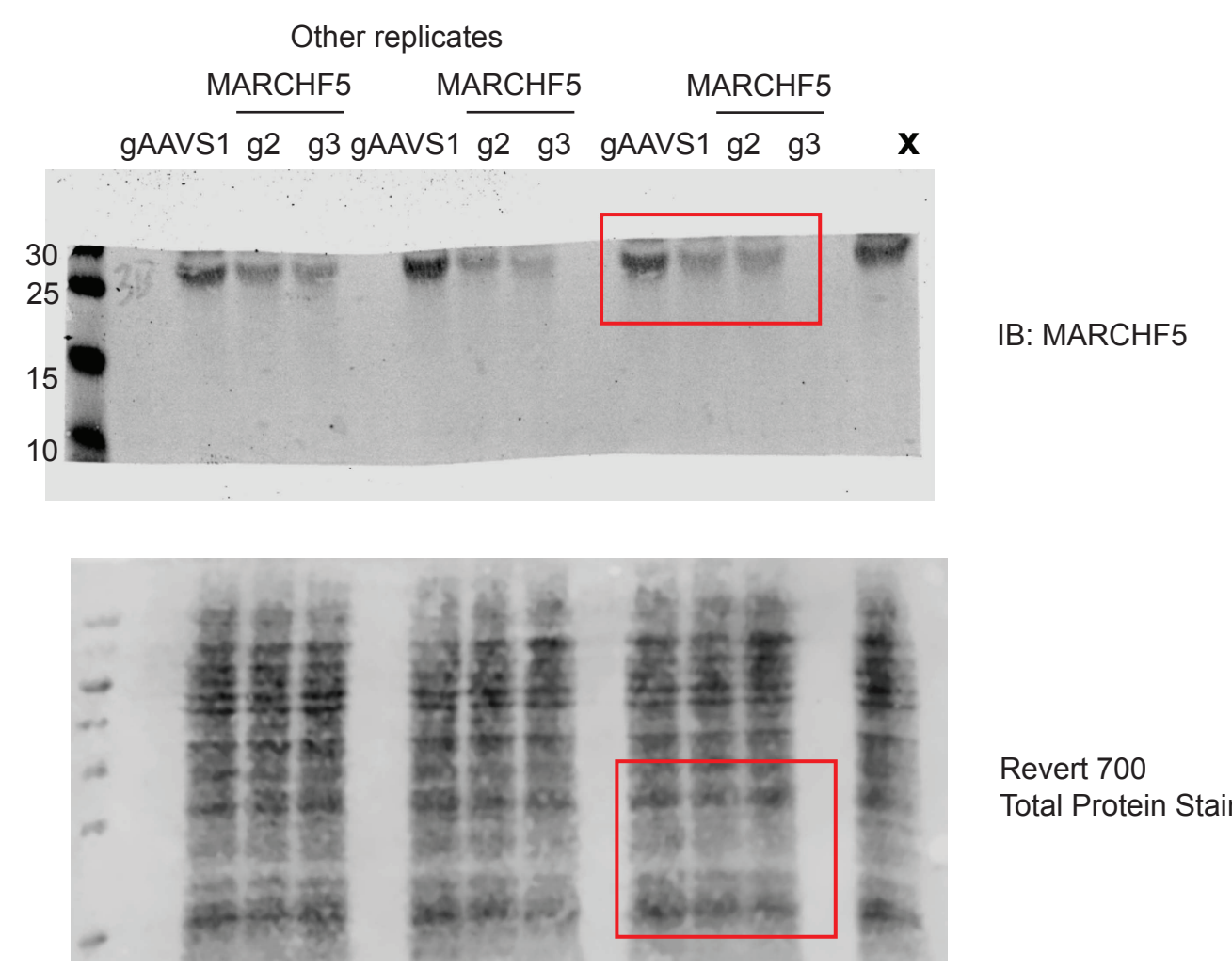

Uncropped blots for Figure 4A

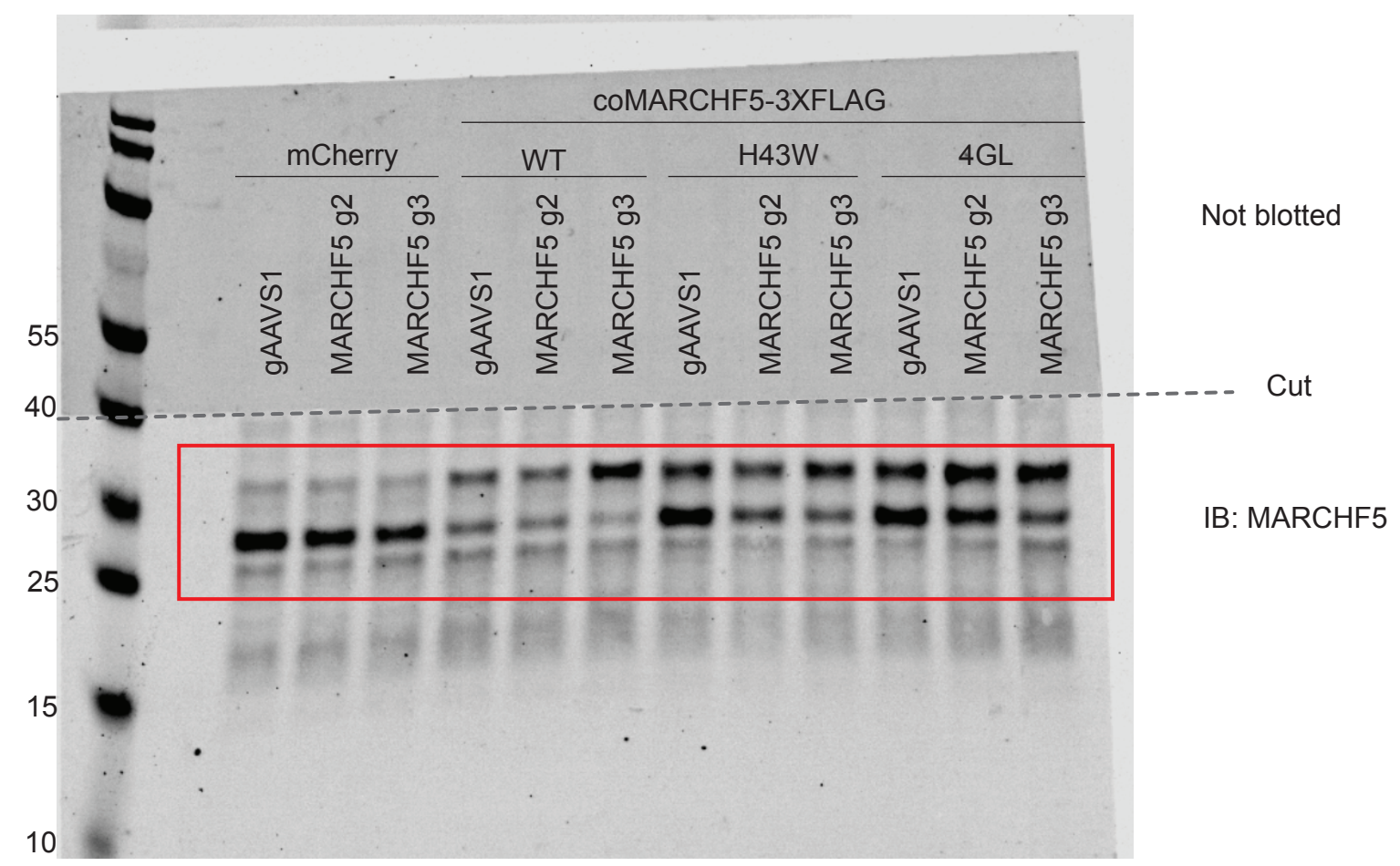

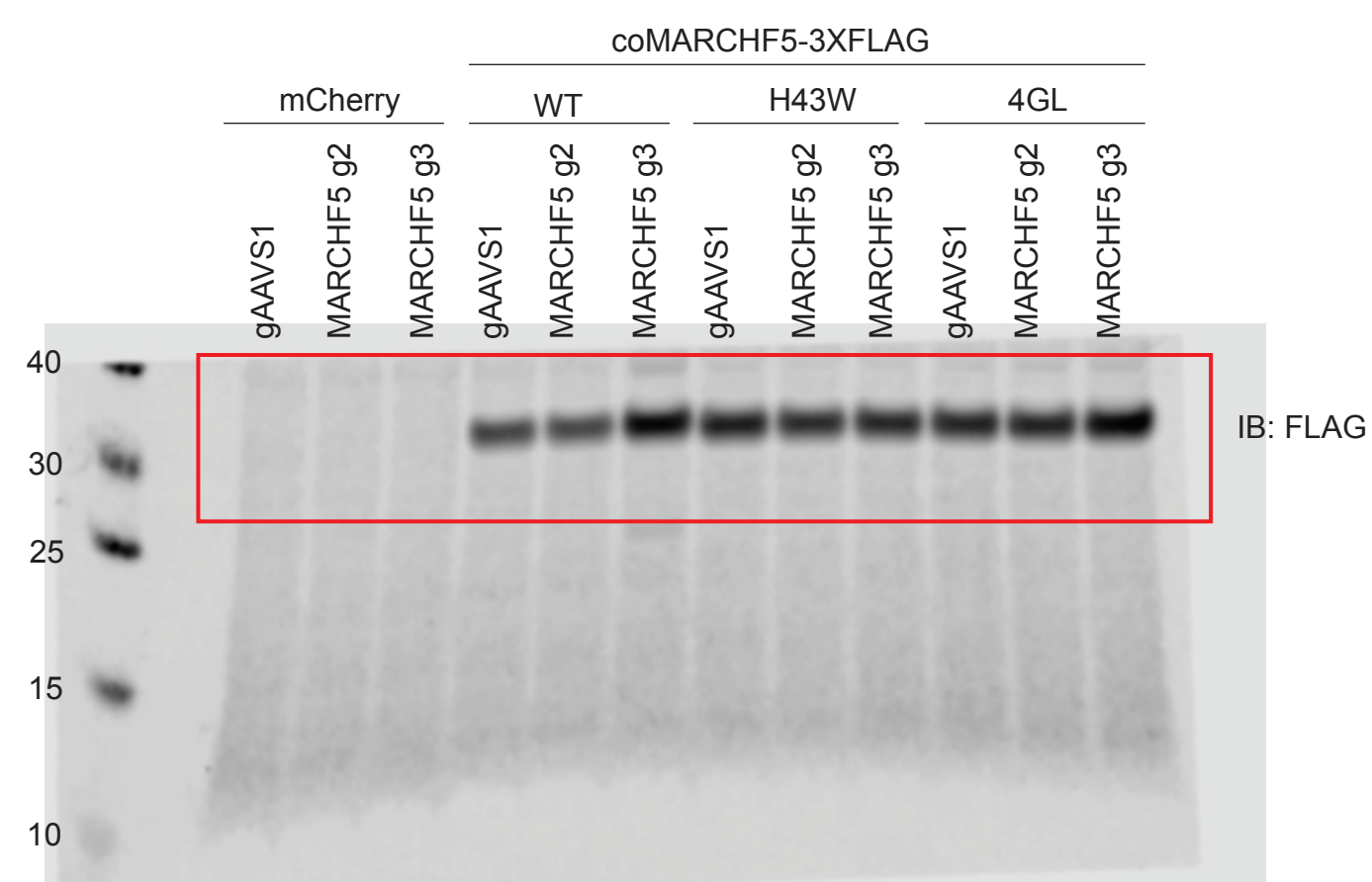

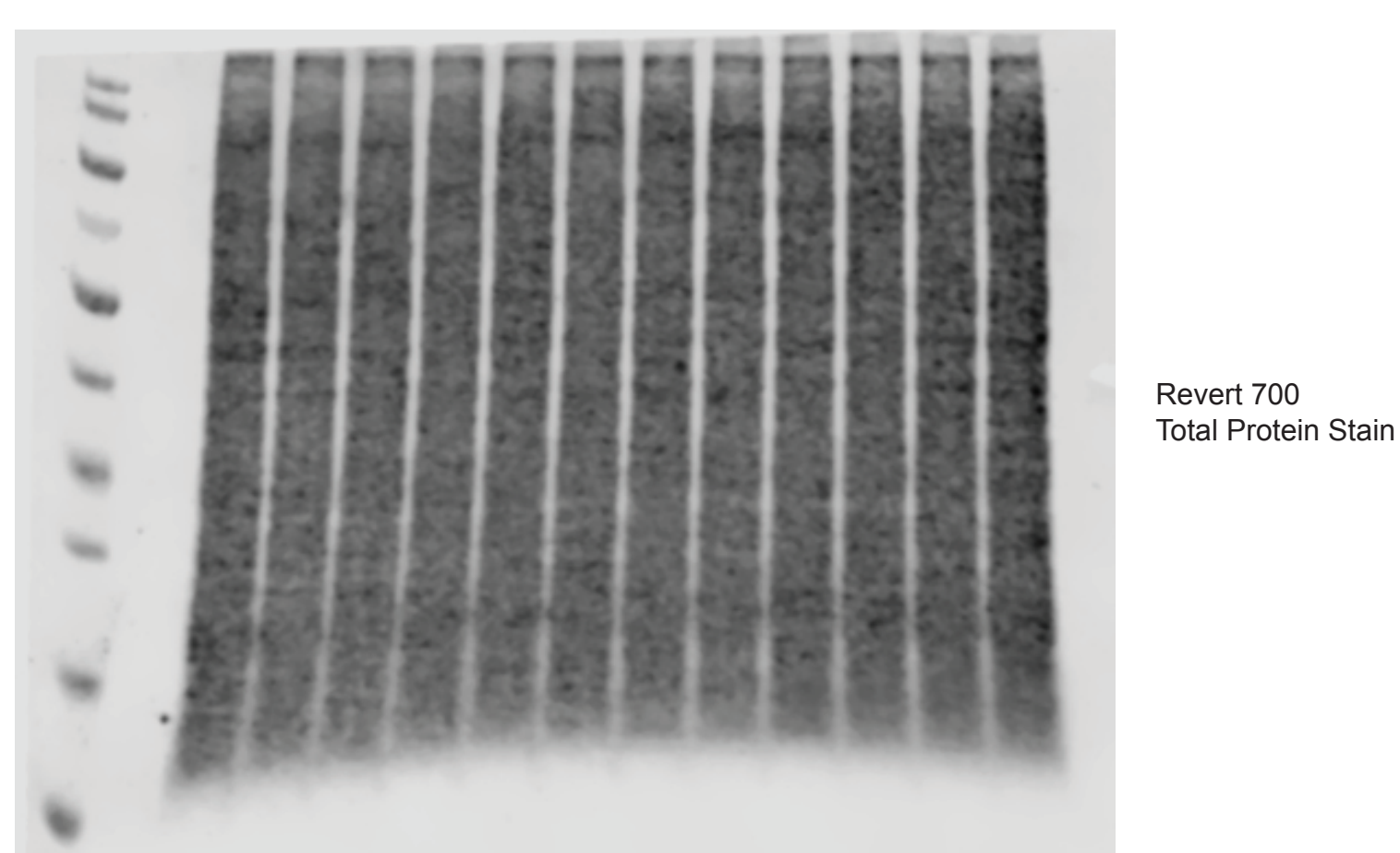

Uncropped blots for Figure 5A

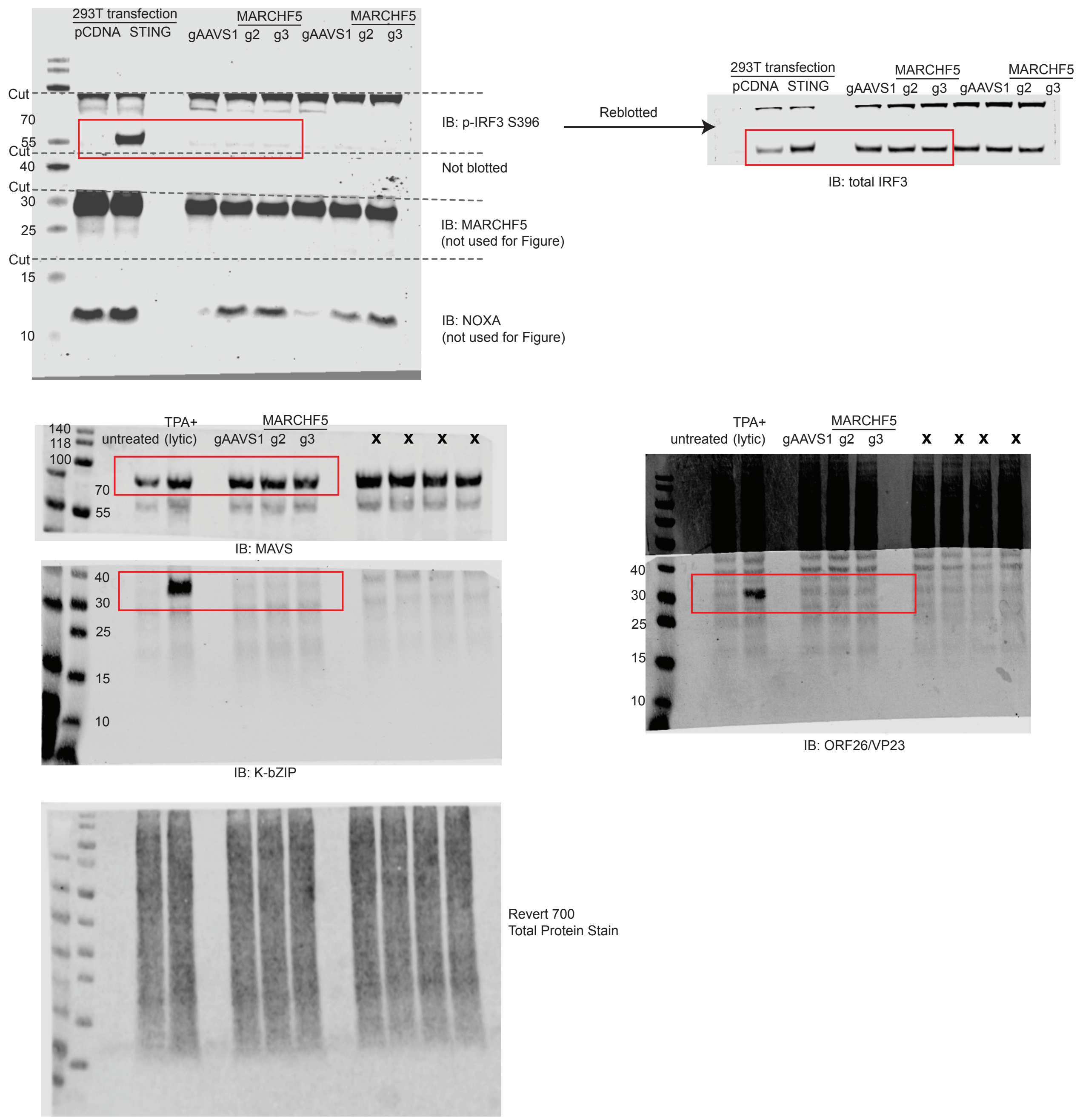

Uncropped blots for Figure 5B

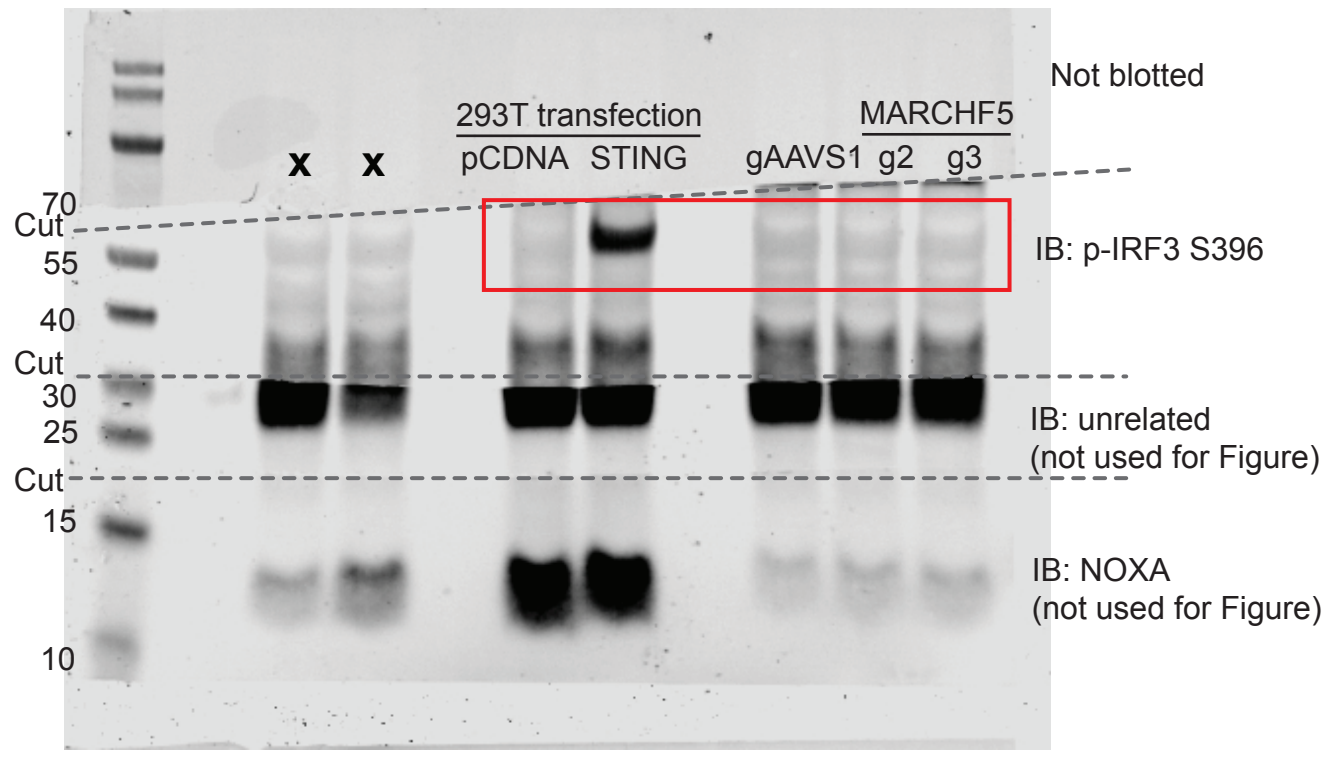

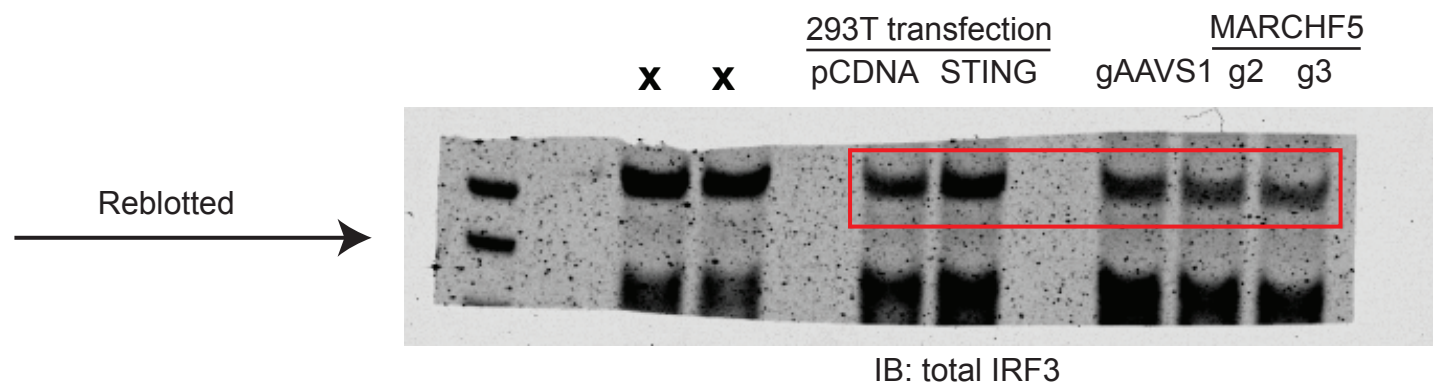

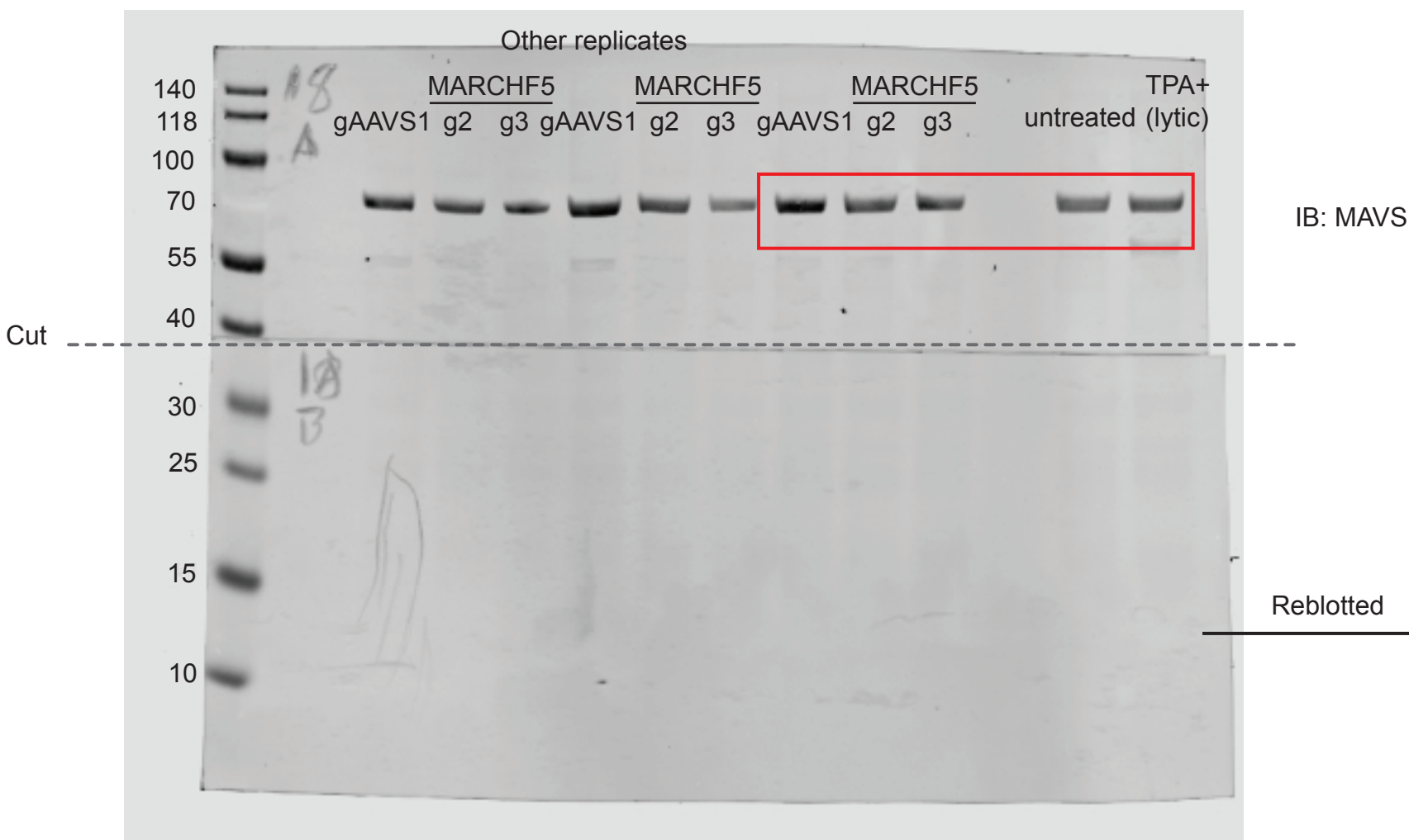

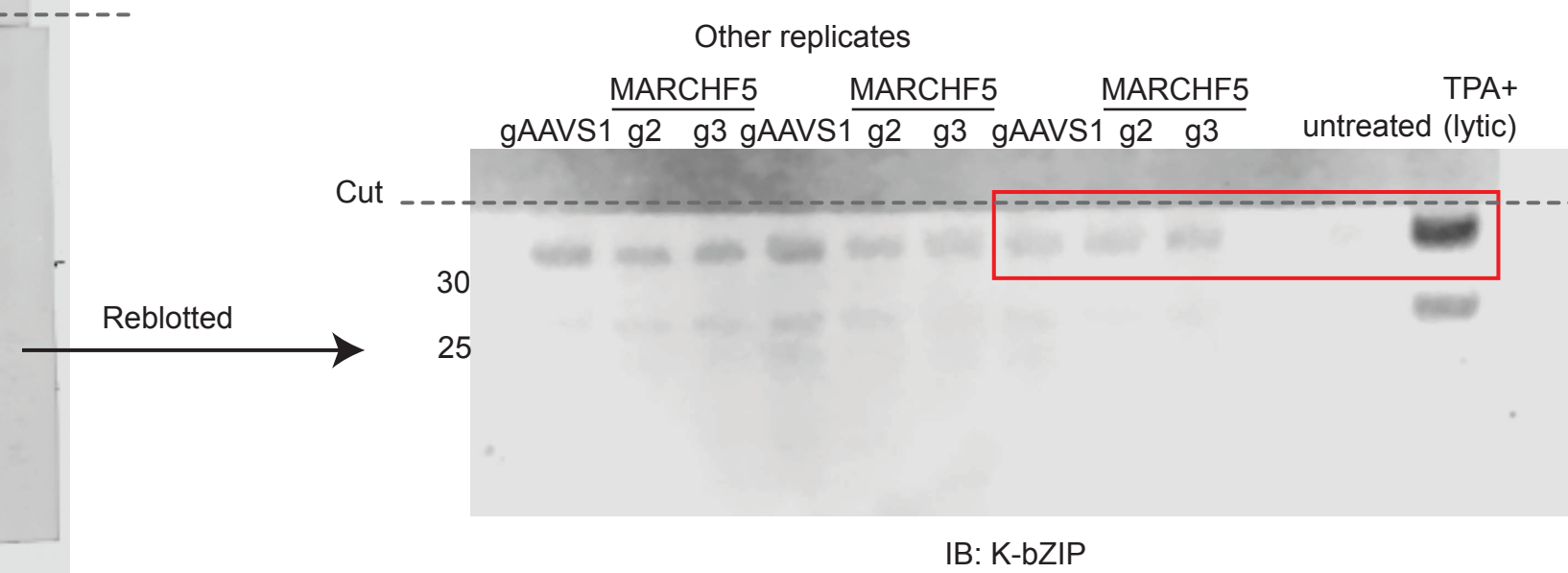

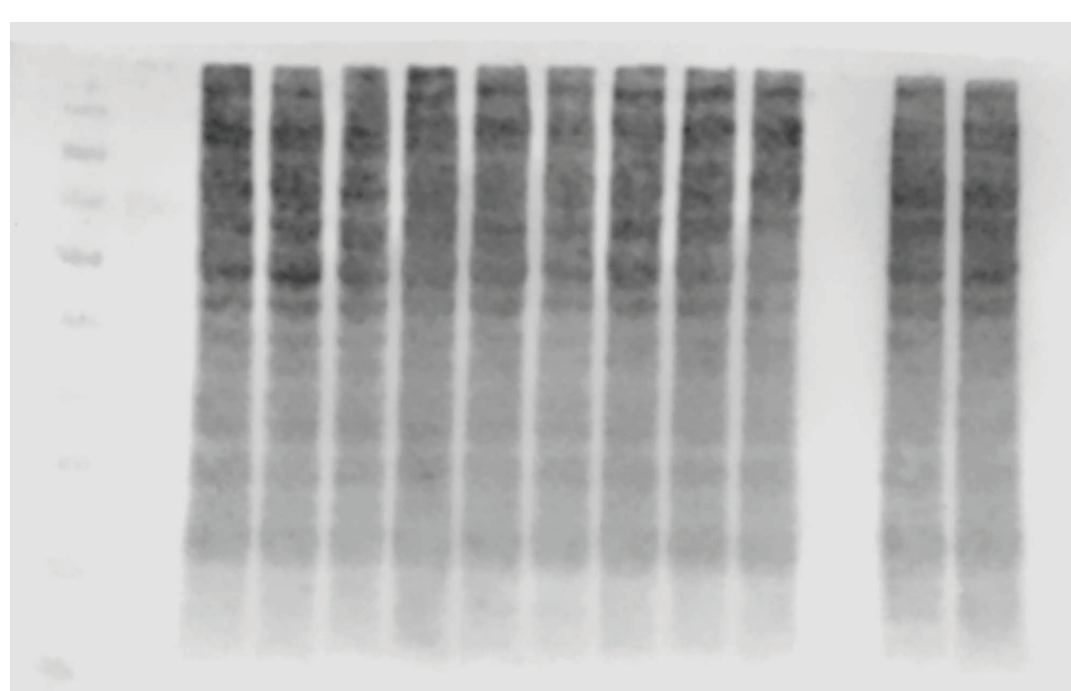

Revert 700
Total Protein Stain

Uncropped blots for Figure 6C

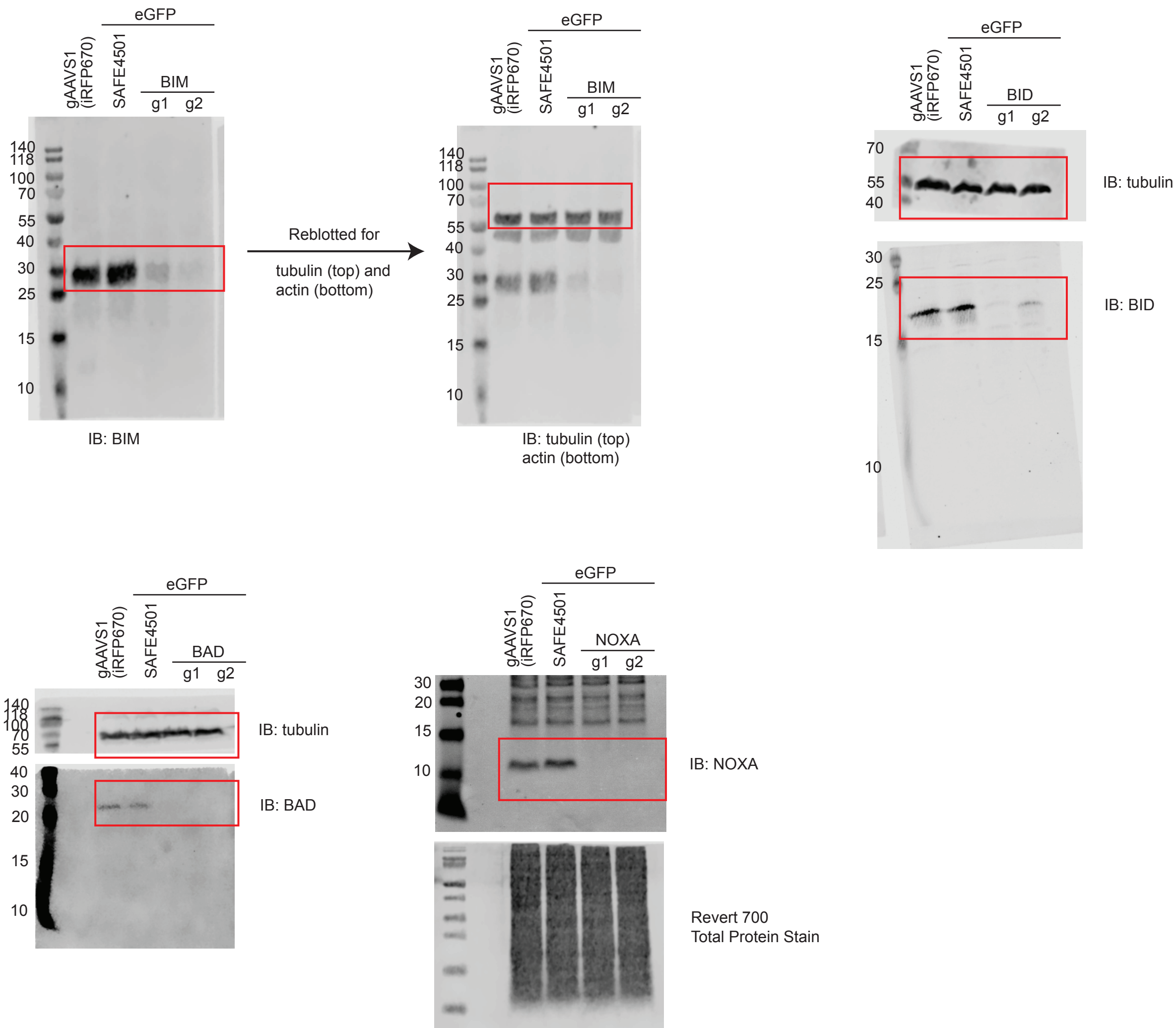

Uncropped blots for Figure 7A

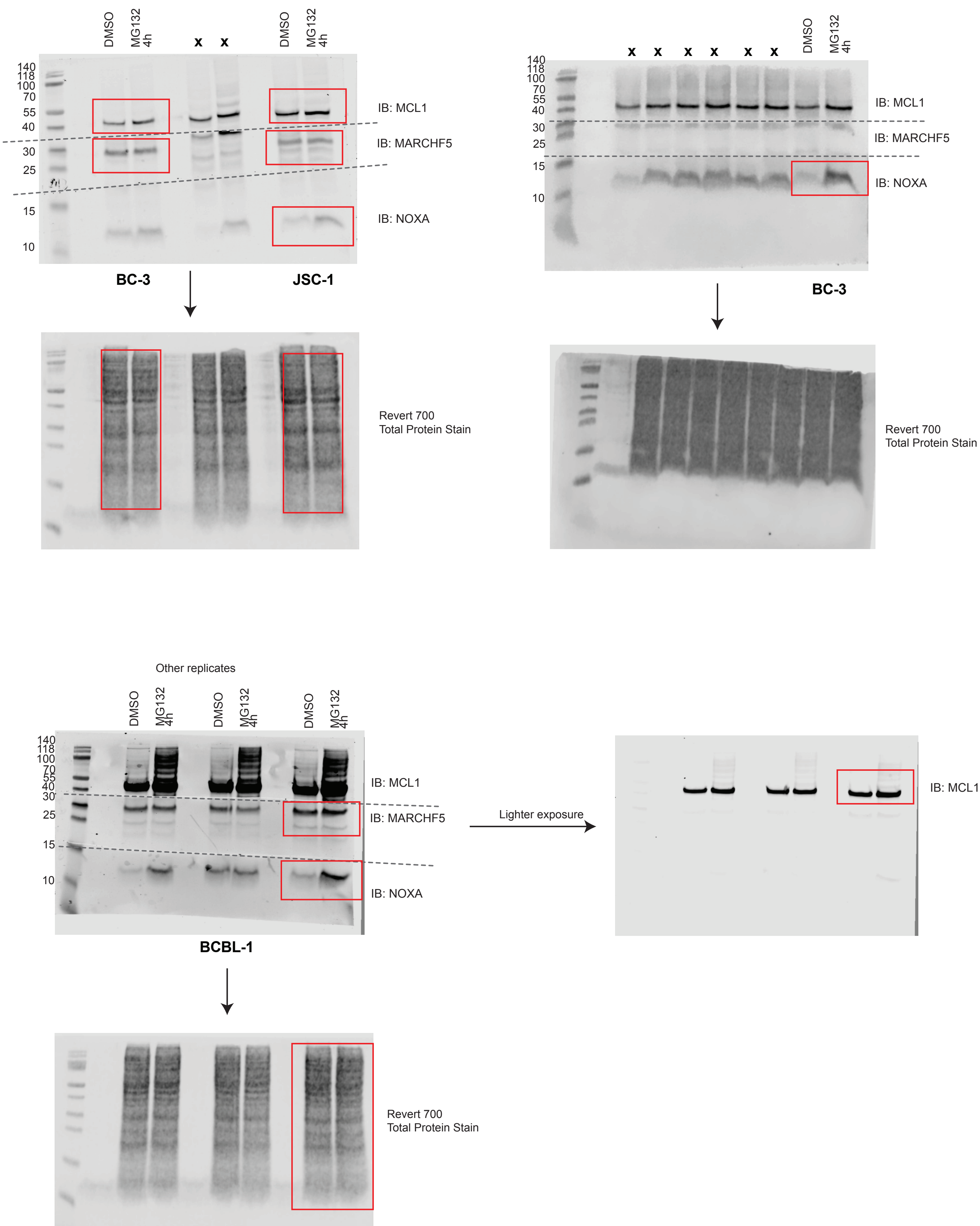

Uncropped blots for Figure 8A (BC-3)

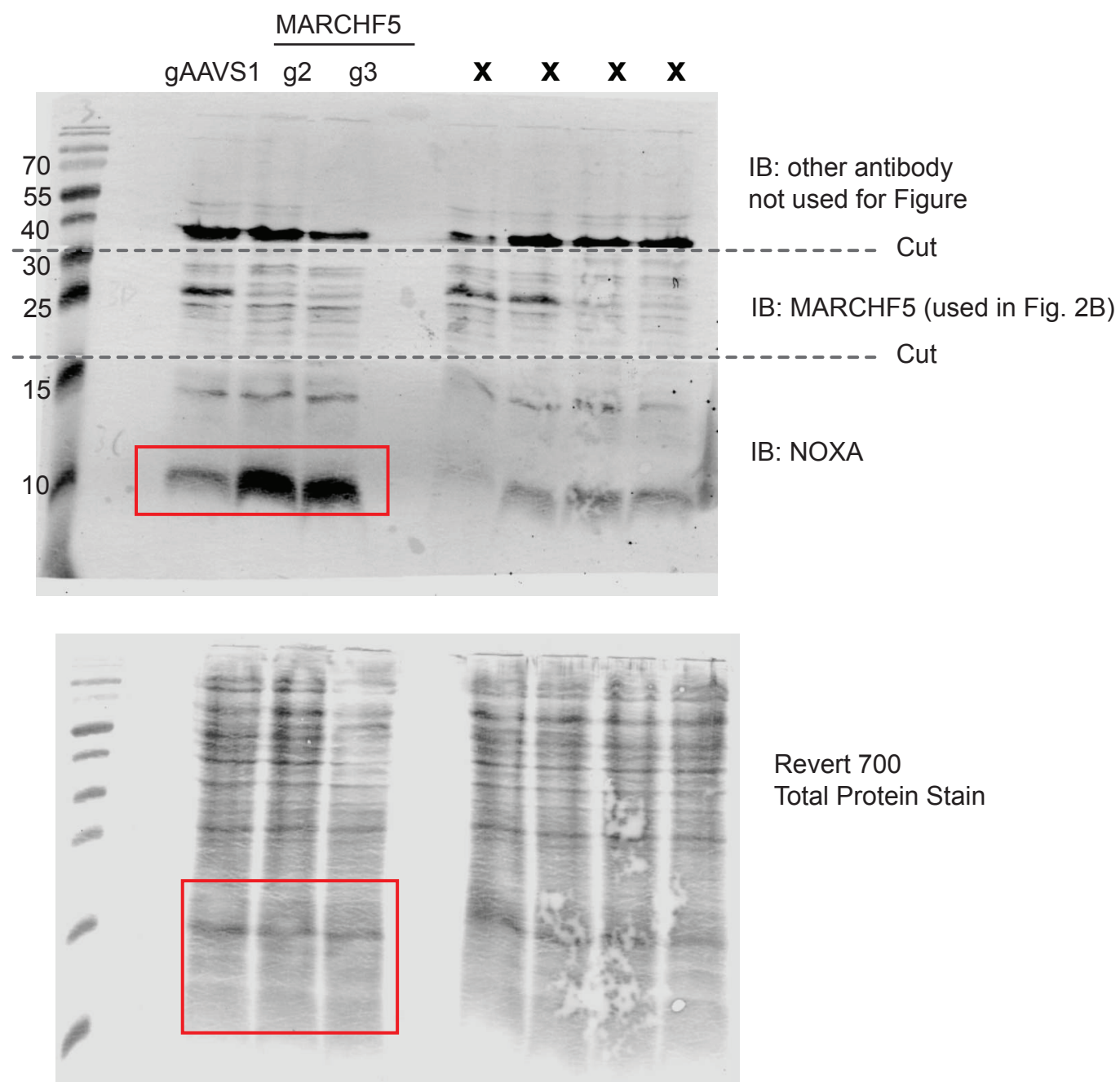

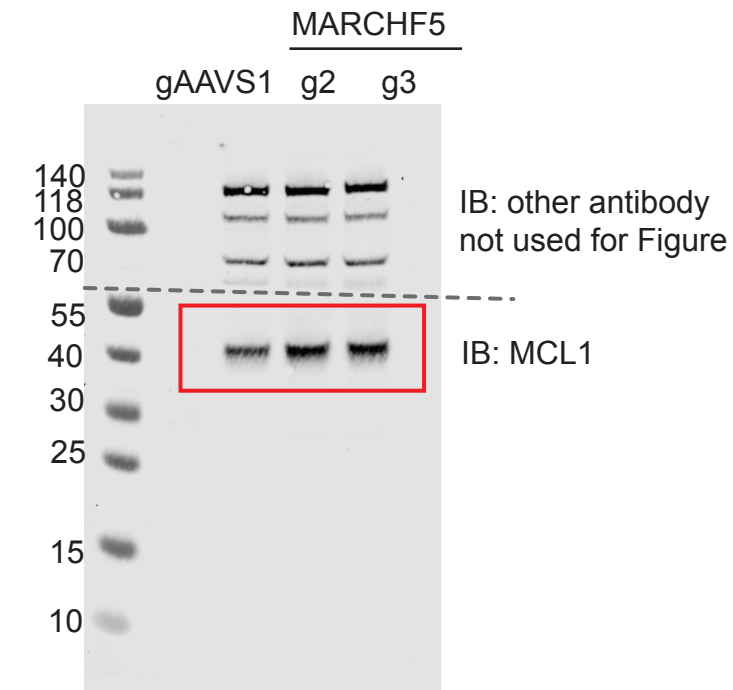

Uncropped blots for Figure 8A (BCBL-1)

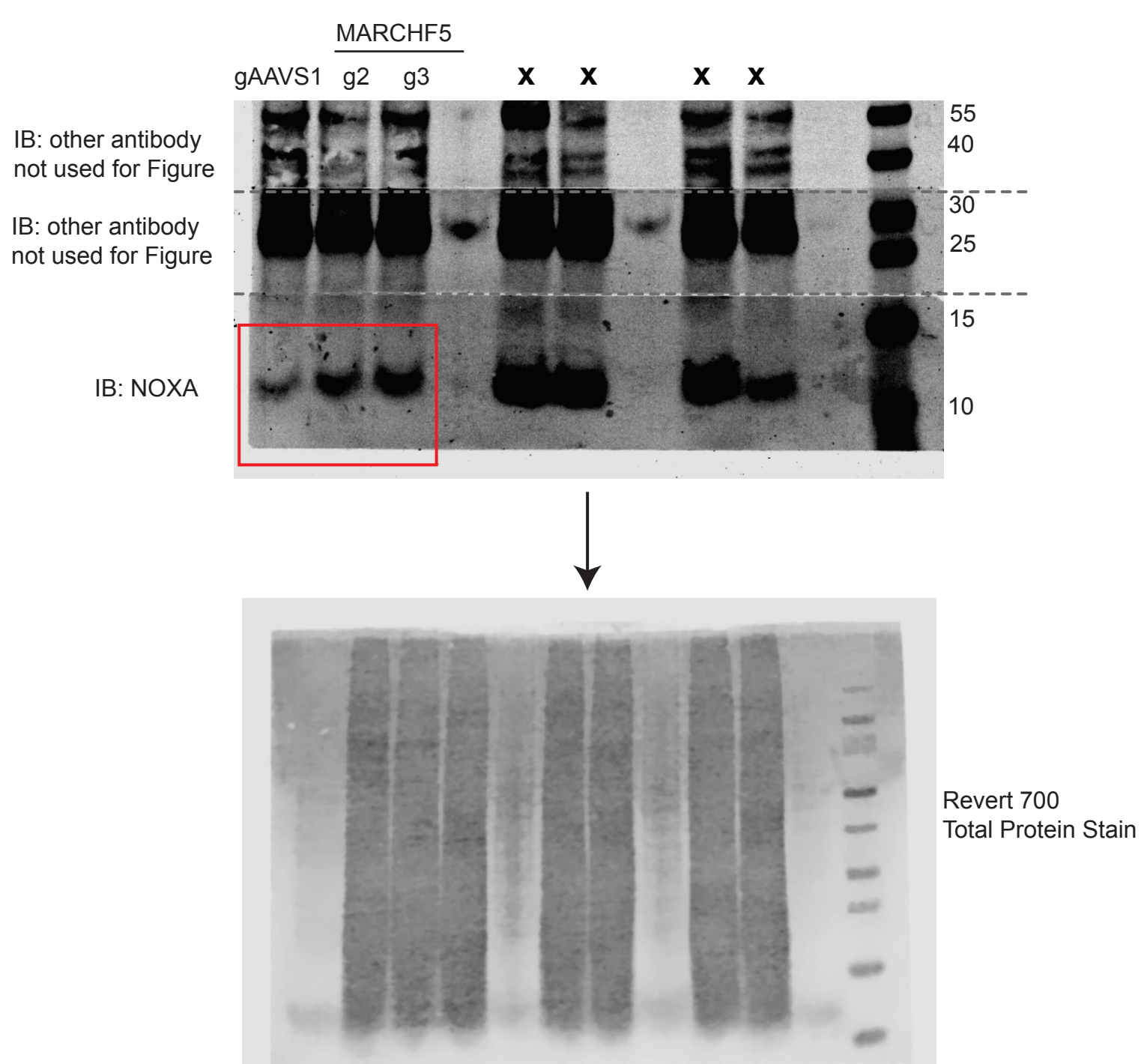

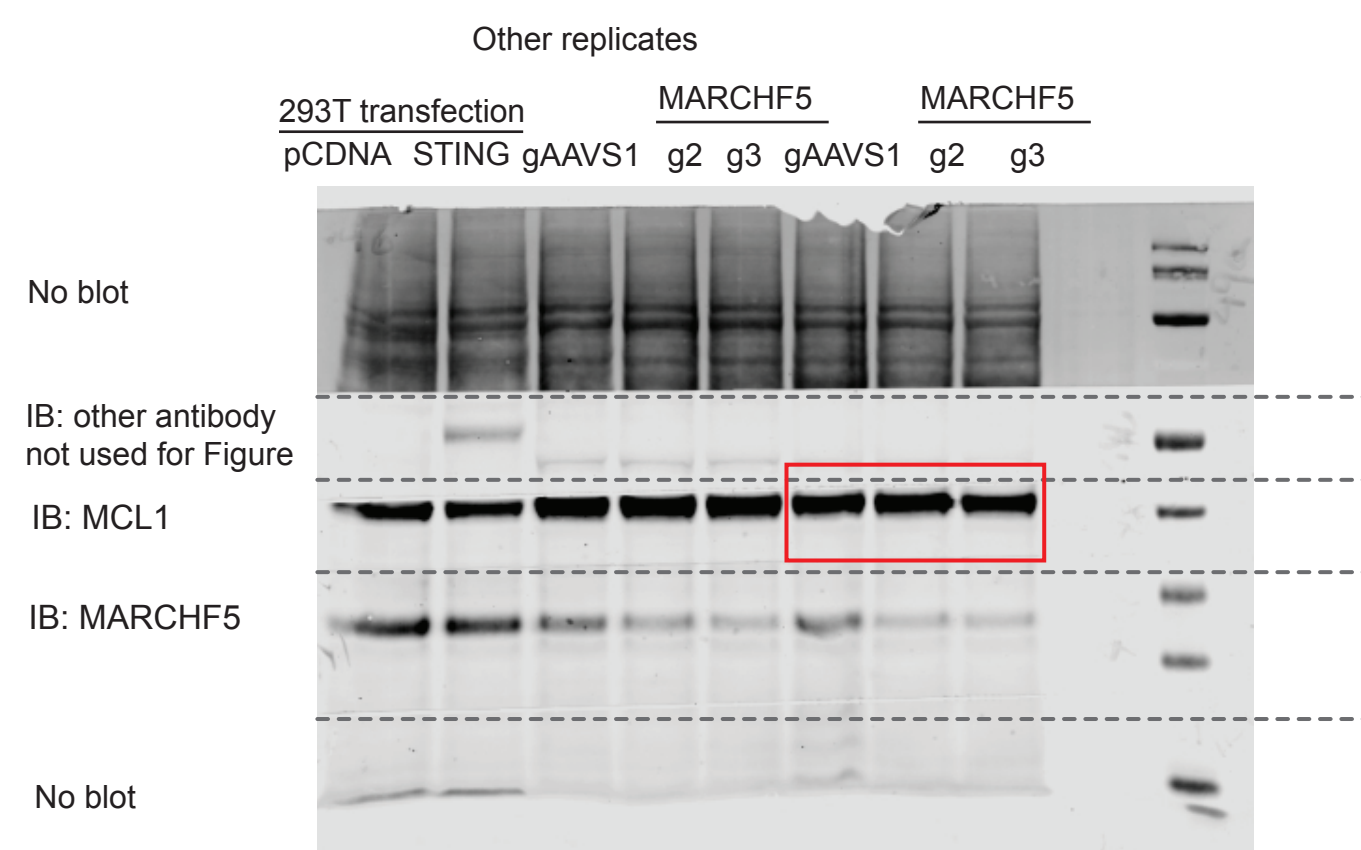

Uncropped blots for Figure 8D

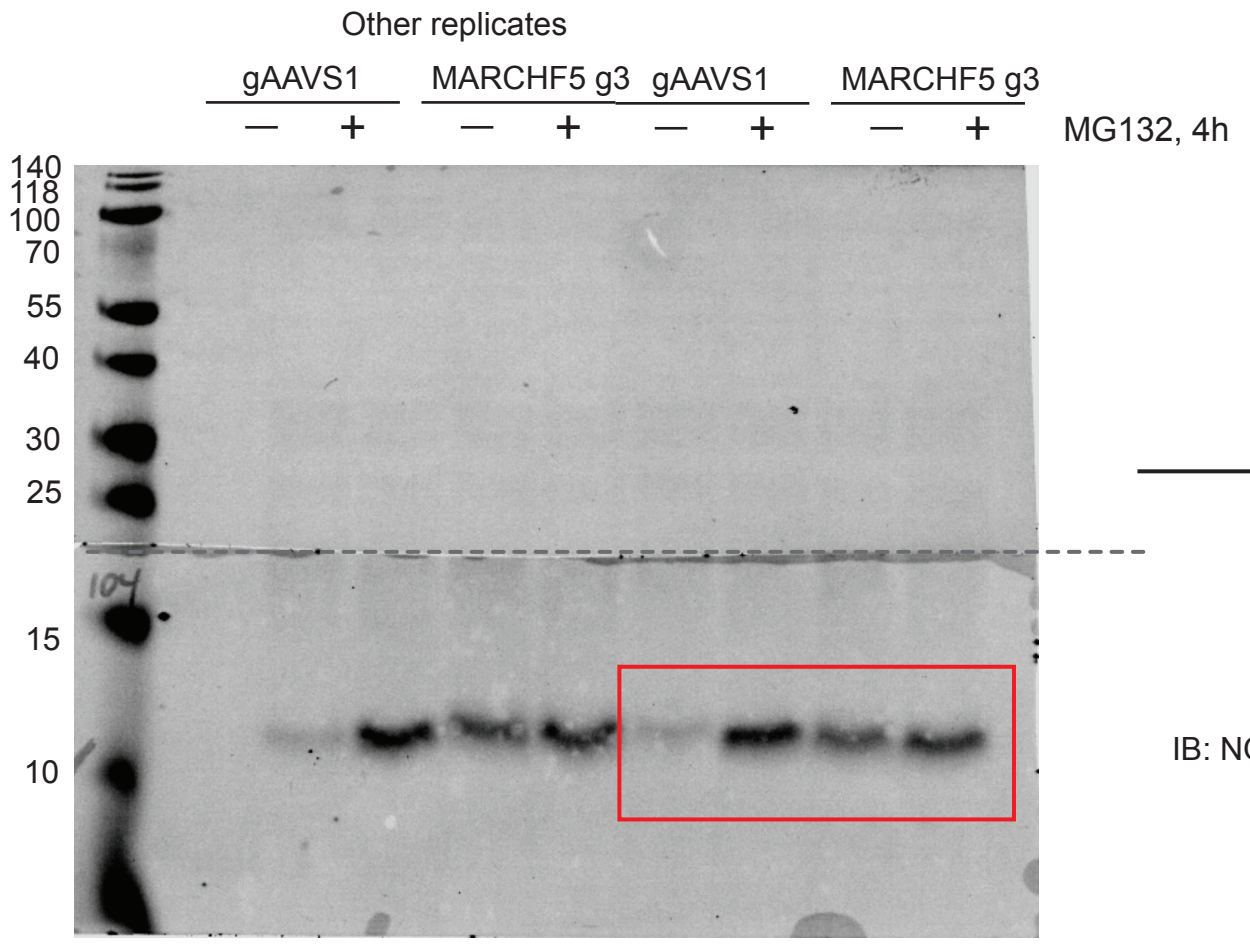

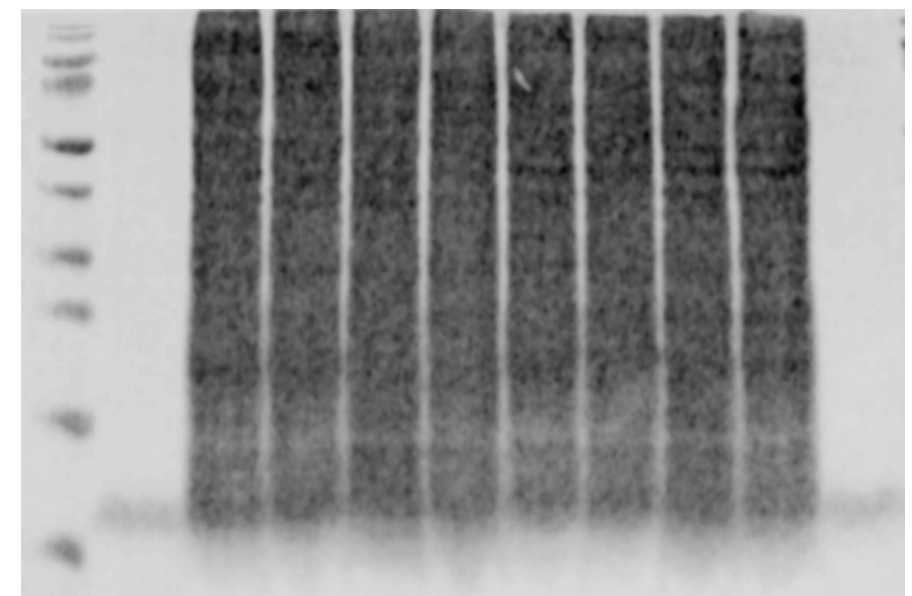

Revert 700
Total Protein Stain

Uncropped blots for Figure 8G

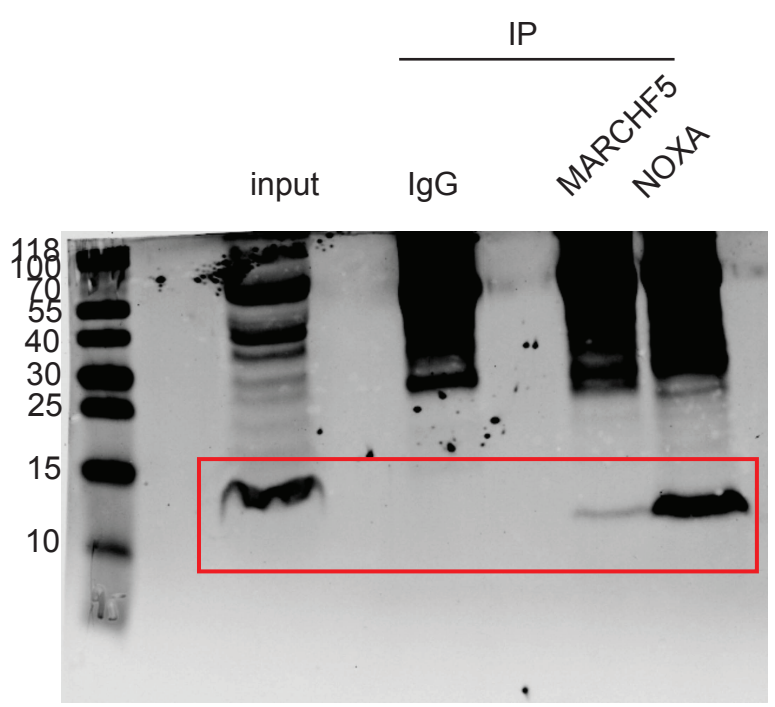

NOXA

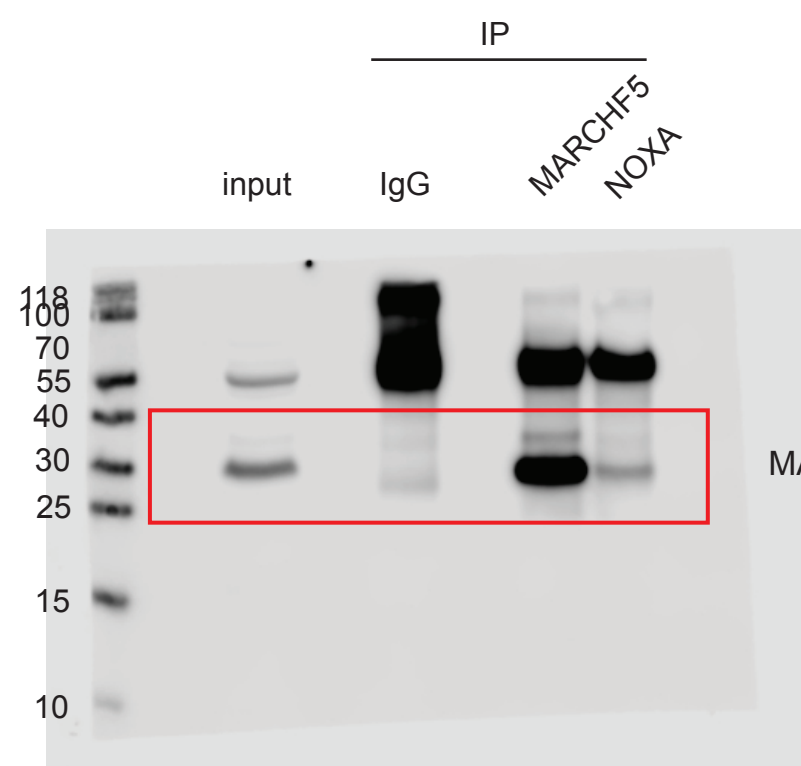

MARCHF5

Uncropped blots for Figure 9B

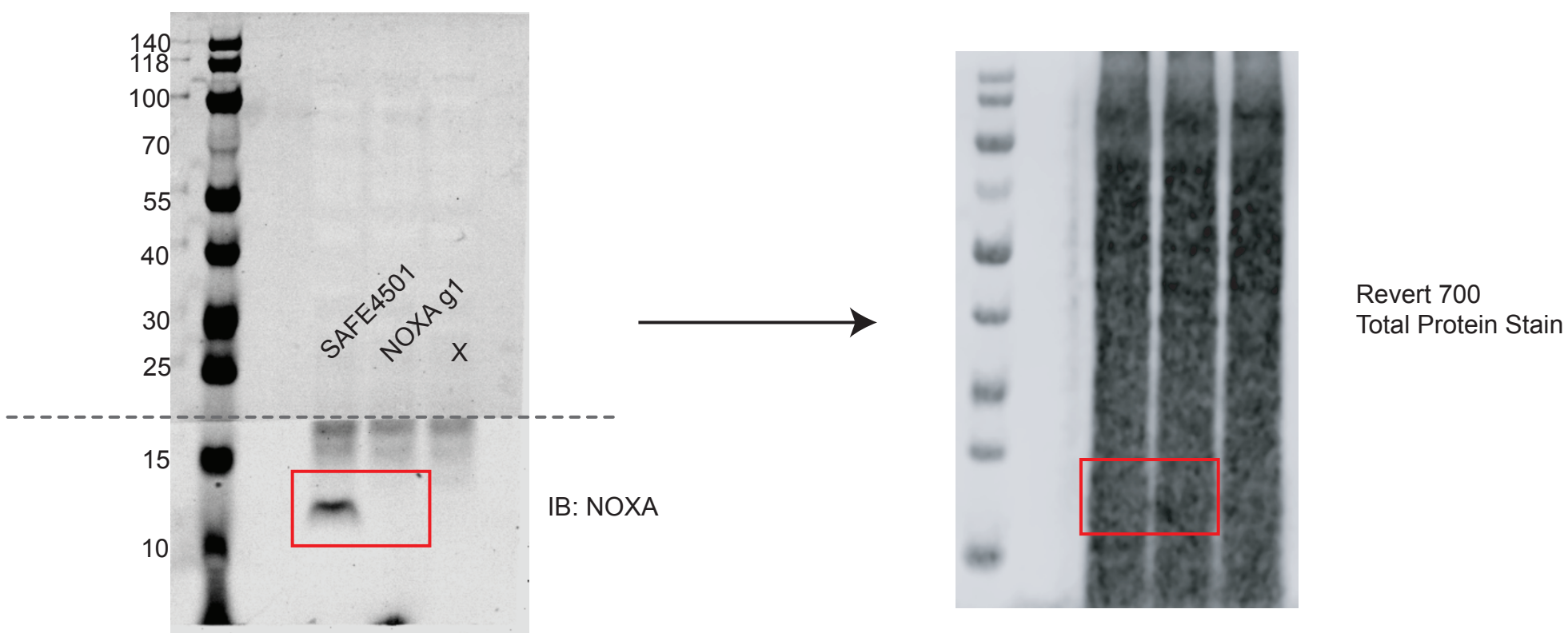

Uncropped blots for Figure 9C

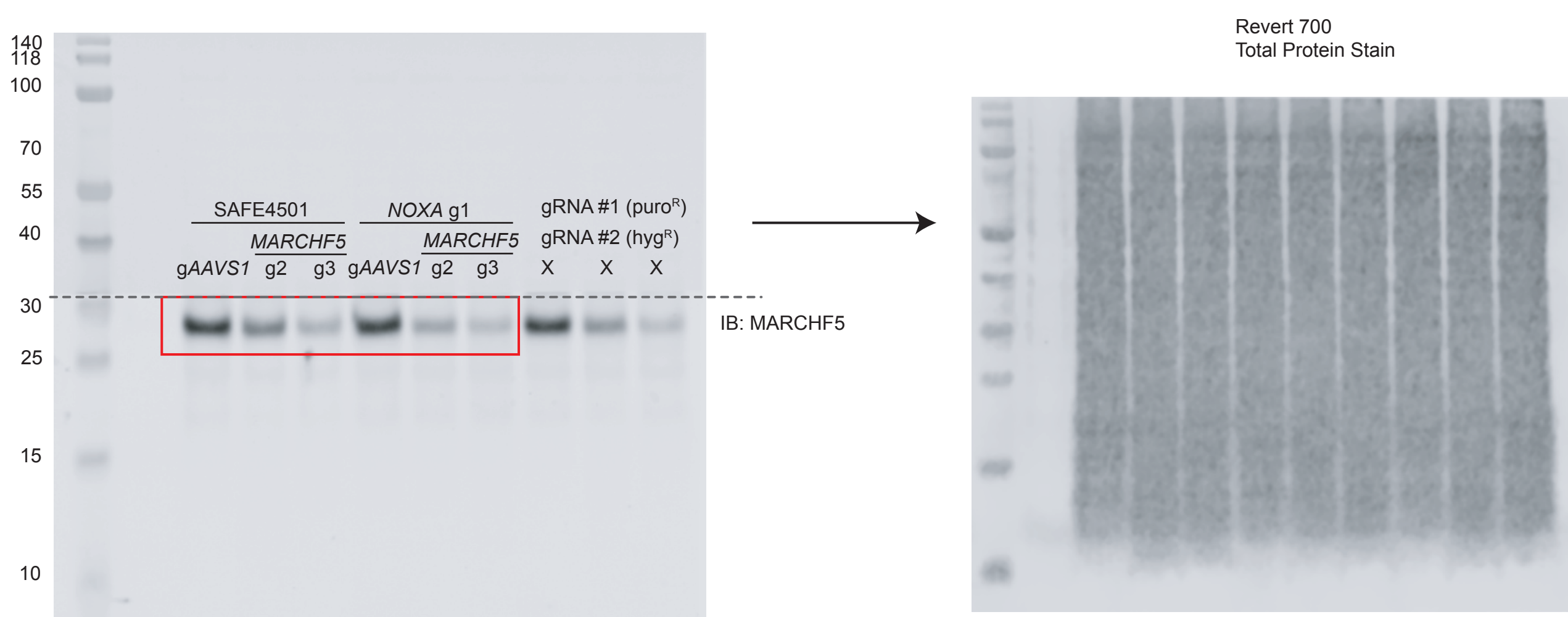

Supplement: Supplementary file 1 [file ppat.1014665.s003.pdf]
